# Potential modifiable factors associated with late-life cognitive trajectories

**DOI:** 10.3389/fneur.2022.950644

**Published:** 2022-08-03

**Authors:** Zimu Wu, Robyn L. Woods, Trevor T. -J. Chong, Suzanne G. Orchard, John J. McNeil, Raj C. Shah, Rory Wolfe, Anne M. Murray, Elsdon Storey, Joanne Ryan

**Affiliations:** ^1^School of Public Health and Preventive Medicine, Monash University, Melbourne, VIC, Australia; ^2^Turner Institute for Brain and Mental Health, Monash University, Melbourne, VIC, Australia; ^3^Department of Neurology, Alfred Health, Melbourne, VIC, Australia; ^4^Department of Clinical Neurosciences, St Vincent's Hospital, Melbourne, VIC, Australia; ^5^Department of Family Medicine and Rush Alzheimer's Disease Center, Rush University Medical Center, Chicago, IL, United States; ^6^Berman Center for Outcomes and Clinical Research, Hennepin Healthcare Research Institute, Minneapolis, MN, United States

**Keywords:** aging, cognitive function, behavior, social support, association, structural equation modeling

## Abstract

**Objective:**

There is variability across individuals in cognitive aging. To investigate the associations of several modifiable factors with high and low cognitive performance.

**Methods:**

Data came from 17,724 community-dwelling individuals aged 65–98 years. Global cognition, verbal fluency, episodic memory, and psychomotor speed were assessed over up to seven years. Group-based multi-trajectory modeling identified distinct cognitive trajectories. Structural equation modeling examined the direct/indirect associations of social/behavioral factors and several chronic conditions with cognitive trajectories.

**Results:**

Seven trajectory subgroups were identified. In the structural equation modeling we compared two subgroups-participants with the highest (14.2%) and lowest (4.1%) cognitive performance with the average subgroup. Lower education, never alcohol intake, and frailty directly predicted increased risk of low performance, and decreased likelihood of high performance. Hypertension (RR: 0.69, 95%CI: 0.60–0.80), obesity (RR: 0.84, 95%CI: 0.73–0.97), diabetes (RR: 0.69, 95%CI: 0.56–0.86) and depression (RR: 0.68, 95%CI: 0.54–0.85) only predicted lower likelihood of high cognitive performance, while dyslipidemia was only associated with low performance (RR: 1.30, 95%CI: 1.07–1.57). Living alone predicted increased risk of low cognitive performance and several comorbidities. Smoking did not predict cognitive trajectories but was associated with increased risk of diabetes, obesity and frailty. Findings were similar when examining the direct associations between modifiable risk factors and all seven cognitive subgroups.

**Conclusions:**

Although several modifiable factors were associated with high performance, and reversely with low performance, this was not observed for obesity, hypertension and dyslipidemia. Further, health behaviors may affect cognitive function indirectly, *via* geriatric conditions. This indicates that strategies to promote healthy cognitive aging, may be distinct from those targeting dementia prevention.

## Introduction

Cognitive changes commonly occur with aging, but there is considerable variability between individuals (1). Most older adults experience varying degrees of cognitive decline, which in some cases may indicate incipient dementia (2). In contrast, some individuals sustain a high level of cognitive function even with advanced age (3). The diversity of late-life cognitive trajectories delineates a spectrum with multiple subgroups having heterogeneous patterns of cognitive aging including high cognitive function over time, average cognitive aging, and low cognitive performance (4, 5).

Evidence regarding modifiable factors related to cognitive impairment is quite well established (2). For example, low education, diabetes, depression, and physical inactivity are all associated with increased risk of lower cognitive performance (2). However, a knowledge gap remains regarding which protective factors predict cognitive resistance with aging, as preliminary evidence suggests that these may be different from those predicting low cognitive performance (6, 7). An important consideration, therefore, is to determine and differentiate the potentially modifiable factors of high cognitive performance and cognitive decline. This will provide novel evidence for not only preventive interventions targeting cognitive decline and dementia, but also promotional strategies for healthy cognitive aging and resistance to cognitive decline.

The influence of some factors on late-life cognitive function remains contentious. For example, previous research observed the associations of hypertension and obesity in mid-life with later dementia, but their effects on cognitive function in late life appear to be much weaker and even opposite (8, 9). Also, alcohol intake has been thought to be a risk factor for cognitive impairment (2), while there is evidence suggesting that the level of intake may an effect modifier (10). Therefore, further research is needed to validate these associations. Moreover, education may not only affect cognitive function directly (11) but is also associated with health behaviors which in turn are risk factors for cognitive decline (12). These potentially modifiable factors, however, might further affect cognitive function *via* comorbidities that are themselves associated with cognitive impairment, such as cardiometabolic disorders, depression, frailty and renal impairment (2, 11, 13, 14), and therefore could have both direct and indirect effects on cognitive function. A better understanding of the complex relationships among these factors and their associations with cognitive function may help formulate social and behavioral interventions.

Using data collected from a cohort of community-dwelling older adults in Australia and the United States, the aims of this study are: (1) use a theoretical framework to investigate the direct associations of modifiable factors with the maintenance of high cognitive performance over time and cognitive decline separately; (2) explore the indirect associations of behavioral/social factors with cognitive function *via* potentially mediating chronic comorbidities.

## Materials and methods

### Study sample

The data used in this study were obtained from the ASPREE (ASPirin in Reducing Events in the Elderly) clinical trial, with details published previously (15). In brief, ASPREE was a randomized placebo-controlled trial to determine the long-term effects of daily low-dose aspirin intake on the health outcomes of older adults. Participants (*n* = 19,114) were adults aged 65+ years (Hispanics/Latino and African American) and 70+ years (all other ethnicities) from Australia and the United States. In Australia, participants were predominantly recruited through general practice. In the United States, a range of community-based methods of recruitment was carried out including clinic-based mailing lists, electronic medical records and media advertisements. At enrolment, participants were without dementia (and with a Modified Mini-Mental State Examination (3MS) score >77), and without established cardiovascular disease, physical activity limitations, or any life-threatening illness. Details of the inclusion and exclusion criteria of the ASPREE clinical trial are presented in Supplementary material.

### Cognitive assessment

Assessment of cognitive function was conducted by trained and accredited staff at baseline, and then at years 1, 3, 4, 5, 6, 7 or close-out visit, covering a maximum of 7 years from 2010 to 2017 (16). The cognitive tests included the 3MS examination for global cognitive function (17), single letter Controlled Oral Word Association Test (COWAT-F) for verbal fluency (18), Hopkins Verbal Learning Test–Revised (HVLT-R) delayed recall task for episodic memory (19), and Symbol-Digit Modalities Test (SDMT) for psychomotor speed (20).

### Baseline characteristics

Information on self-reported sociodemographic characteristics included age, gender, ethnicity (Australian White/US White/African American/Hispanic or Latino/Other), and years of education) was obtained at baseline. Health behaviors were also reported, including smoking status, alcohol intake, and living alone. Height, body weight and blood pressure were measured by physical examination. Blood and urinary tests were conducted to measure total cholesterol, low-density lipoprotein (LDL), fasting glucose, estimated glomerular filtration rate (eGFR), and urinary albumin to creatinine ratio (ACR). Chronic health conditions and current medications used were also obtained *via* self-report and medical records. The following comorbidities were considered: obesity, defined as body mass index ≥ 30 kg/m2; diabetes, defined from self-report or fasting glucose ≥ 126 mg/dL (≥7 mmol/L) or on treatment for diabetes; hypertension, defined as on treatment for high BP or BP > 140/90 mmHg; dyslipidemia defined as those taking cholesterol-lowering medications or serum cholesterol ≥212 mg/dL (≥5 mmol/L; Australia) and ≥240 mg/dL (≥6.2 mmol/L) or LDL > 160 mg/dL (>4.1 mmol/L); chronic kidney disease defined as eGFR < 60 ml/min/1.73 m^2^ or ACR ≥ 3 mg/mmol); depressive symptoms were assessed using the 10-item Center for Epidemiological Studies-Depression Scale (CES-D-10) as a score of 8 or higher (21); and pre-frailty/frailty was assessed using the modified Fried criteria (including being underweight, weak grip strength, exhaustion, slow walking speed and low physical activity) (22, 23).

### Statistical analysis

Group-based multi-trajectory modeling was used to identify latent classes of cognitive trajectories across the four cognitive tests. Group-based trajectory modeling (GBTM) is a specialized application of finite mixture modeling, which identifies a mixture of heterogeneous clusters of individuals following homogenous trajectories, based on the complex distribution of the longitudinal sequence of an indicator (24). Group-based multi-trajectory modeling is an extension of the univariate GBTM. This technique jointly estimates the group-based trajectories for multiple related indicators that evolve simultaneously and analyses their connections by linking probabilities (25). Longitudinal cognitive data collected at up to seven timepoints were used to define cognitive trajectories. Participants included in trajectory modeling were required to have data of the four cognitive tests at baseline and at least one subsequent timepoint. The pre-specified model selection criteria were summarized in Supplementary Table 1. To compare individuals according to their cognitive performance, we aimed a priori to compare three subgroups of participants with hierarchical cognitive performance–(1) high performers with the highest intercepts and least declines for all cognitive tests; (2) average performers; (3) low performers with the lowest intercepts and lowest scores over follow-up or steepest slopes across the four tests. If more than three classes were identified as the optimal solution, the highest and the lowest classes were both compared against the central class based on the intercepts and slopes of the trajectories, permitting interpretable comparison without compromising on model fit and model precision. However, in such a case, sensitivity analyses including all classes was also undertaken, to ensure robustness of findings.

**Figure 1:**
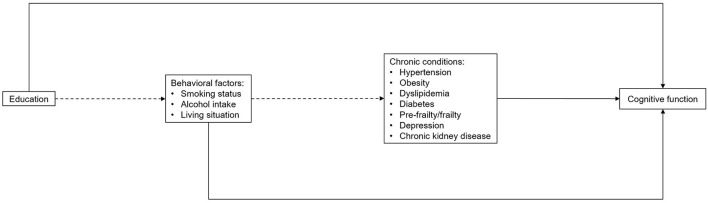
Theoretical framework of structural equation modeling, including paths sequentially from education to behavioral factors, chronic conditions and cognitive function. Solid arrows refer to the direct associations and dashed arrows refer to the indirect associations with cognitive trajectory subgroups. Age, gender and ethnicity were also included in the full model with arrows to all other variables (not shown).

We applied a structural equation model (SEM) based on a theoretical framework (Figure 1) to analyze the associations of potential modifiable factors with cognitive trajectory subgroups. Direct associations are presented as solid arrows, and indirect associations as dashed arrows. Each arrow represents either a binary or a multinomial logit model, depending on the targeted variable. All variables of modifiable factors were introduced into the analysis at this stage. The path analysis of SEM allows the examination of complex associations among a set of variables including the direct and indirect associations of these variables on the outcome given a set of assumptions (26). Therefore, most variables were treated as both dependent and independent variables, depending on the path. To simplify the analysis and improve the interpretability, all variables of modifiable factors were dichotomized. We examined the direct associations of all potentially modifiable factors with cognitive performance based on previous studies and available data in the ASPREE study (2, 27). Prior evidence also suggested that educational level can influence health behaviors, and health behaviors could further affect the risk of a number of common geriatric diseases (2, 11, 12). Therefore, the associations of education with the three behavioral factors (smoking status, alcohol intake, living alone) were also examined, as well as the associations of living alone with smoking status and alcohol intake, and the indirect associations of the three behavioral factors with cognitive function *via* chronic conditions.

Although the focus of this study was to identify modifiable factors, we adjusted for age, gender and ethnicity in the model, given their established associations with cognitive function and to remove confounding from these sources. Participants with missing values in any of these variables were excluded. The baseline characteristics of ASPREE participants were compared between those who were included and those who were excluded from the current analysis. Sensitivity analyses were conducted to test the robustness of the findings, specifically: (a) by modeling high (class 1 vs. class 4) and low cognitive performance (class 7 vs. class 4) as separate constructs in the path analysis; (b) by using multinomial logistic regression to ascertain the direct associations of all variables, along with subgroup analyses within participants of major ethnicities and across educational levels; (c) by including all participants of trajectory modeling in the path analysis. All statistical analyses were conducted using Stata version 16.0 (Stata Corp., College Station, Texas, USA). The ASPREE clinical trial was conducted according to the guidelines of the International Conference on Harmonization Good Clinical Practice. All participants offered written informed consent. The current study was approved by the Monash University Human Research Ethics Committee.

## Results

After excluding individuals with incomplete cognitive data at baseline or at all follow-up visits in any of the four tests, 17,724 participants were included in the trajectory modeling. Compared to those excluded, these participants were more likely to be male and current alcohol drinkers, while less likely to live alone or be current smokers, and with lower prevalence of chronic conditions (Supplementary Table 1). A total of seven classes with hierarchically distinct cognitive performance were identified (Supplementary Table 2; Supplementary Figure 1). Based on their intercepts and slopes (Supplementary Table 3), a group of high performers with the highest trajectories across the four cognitive tests (class 1, 14.2%), and low performers with the lowest trajectories across the test (class 7, 4.1%), were identified. These groups were compared with the medium class (class 4, 21.6%) representing average-performers in the structural equation modeling (SEM) analysis.

The baseline characteristics of the study participants overall and in the three subgroups being compared in the SEM analysis, is shown in Table 1. Compared with the average and low performers, the high performers were younger, more likely to be females, US White, tertiary educated, and ever (current or former) alcohol drinkers. Additionally, they were less likely to live alone, be ever smokers, or have any of the chronic comorbidities. However, there was a higher proportion of individuals with dyslipidemia in the high-performer group compared with the other two groups.

**Table 1:** Baseline characteristics of participants included in the study (*N* = 17,724).

	**Subgroup (** ***N*** **, %)**				
	**Total** **(17,724, 100%)**	**High performers** **(2,512, 14.2%)**	**Average performers** **(3,835, 21.6%)**	**Low performers** **(720, 4.1%)**	***X^2^* test;** ***P-*value**
Age, years					<0.001
65–69*	483 (2.7)	66 (2.6)	115 (3.0)	13 (1.8)	
65–74	9,945 (56.1)	1,890 (75.2)	1,863 (48.6)	229 (31.8)	
75–84	6,658 (37.6)	542 (21.6)	1,699 (44.3)	377 (52.4)	
≥85	638 (3.6)	14 (0.6)	158 (4.2)	101 (14.0)	
Gender					<0.001
Men	7,764 (43.8)	745 (29.7)	1,846 (48.1)	396 (55.0)	
Women	9,960 (56.2)	1,767 (70.3)	1,989 (51.9)	324 (45.0)	
Ethnicity					<0.001
AU White	15,249 (86.0)	2,116 (84.2)	3,349 (87.3)	606 (84.2)	
US White	1,020 (5.8)	262 (10.4)	138 (3.6)	28 (3.9)	
African American	765 (4.3)	62 (2.5)	181 (4.7)	46 (6.4)	
Hispanic/Latino	434 (2.5)	39 (1.6)	126 (3.3)	24 (3.3)	
Others	256 (1.4)	33 (1.3)	41 (1.1)	16 (2.2)	
Education, years					<0.001
≤ 12	7,974 (45.0)	596 (23.7)	2,106 (54.9)	438 (60.8)	
>12	9,750 (55.0)	1,916 (76.3)	1,729 (45.1)	282 (39.2)	
Living alone at home					<0.001
Yes	5,732 (32.3)	768 (30.6)	1,261 (32.9)	292 (40.6)	
No	11,992 (67.7)	1,744 (69.4)	2,574 (67.1)	428 (59.4)	
Ever smoker					0.009
Yes	7,835 (44.2)	1,030 (41.0)	1,745 (45.5)	321 (44.6)	
No	9,889 (55.8)	1,482 (59.0)	2,090 (54.5)	399 (55.4)	
Ever alcohol intake					<0.001
Yes	14,689 (82.9)	2,184 (86.9)	2,856 (81.8)	561 (77.9)	
No	3,035 (17.1)	328 (13.1)	686 (17.9)	159 (22.1)	
Hypertension					<0.001
Yes	13,123 (74.0)	1,668 (66.4)	2,963 (77.3)	551 (76.5)	
No	4,601 (26.0)	844 (33.6)	872 (22.7)	169 (23.5)	
Dyslipidemia					<0.001
Yes	11,585 (65.4)	1,737 (69.2)	2,448 (63.8)	472 (65.6)	
No	6,139 (34.6)	775 (30.8)	1,387 (36.2)	248 (34.4)	
Obesity					<0.001
Yes	5,203 (29.5)	670 (26.8)	1,187 (31.1)	196 (27.4)	
No	12,448 (70.5)	1,835 (73.2)	2,630 (68.9)	520 (72.6)	
Diabetes					<0.001
Yes	1,869 (10.5)	171 (6.8)	444 (11.6)	107 (14.9)	
No	15,855 (89.5)	2,341 (93.2)	3,391 (88.4)	613 (85.1)	
Pre-frailty/frailty					<0.001
Yes	7,134 (40.3)	685 (27.3)	1,690 (44.1)	451 (62.4)	
No	10,590 (59.7)	1,827 (72.7)	2,145 (55.9)	269 (37.4)	
Depression					<0.001
Yes	1,702 (9.6)	170 (6.8)	385 (10.0)	96 (13.3)	
No	16,019 (90.4)	2,342 (93.2)	3,449 (90.0)	624 (86.7)	
Chronic Kidney disease					<0.001
Yes	4,331 (26.2)	481 (20.4)	1,018 (28.3)	238 (35.4)	
No	12,166 (73.8)	1,876 (79.6)	2,575 (71.7)	435 (64.6)	

* Only includes U.S. African American or Hispanic/Latino participants, who were eligible to enroll from 65 years or above (all other participants needed to be 70 years or above to be recruited). (1) hypertension was defined as on treatment for high BP or BP >140/90 mmHg at study entry; (2) dyslipidemia was defined as those taking cholesterol-lowering medications or serum cholesterol ≥212 mg/dL (≥5 mmol/L; Australia) and ≥240 mg/dL (≥6.2 mmol/L; U.S.) or LDL > 160 mg/dL (>4.1 mmol/L); (3) obesity was defined as body mass index ≥30; (4) diabetes was defined from self-report or fasting glucose ≥126 mg/dL (≥7 mmol/L) or on treatment for diabetes; (5) frailty was defined using the adapted Fried frailty criteria, including being underweight, weak grip strength, exhaustion, slow walking speed and low physical activity, with pre-frail including anyone with 1 or 2 criteria and Frail as anyone with three or more criteria (22); (6) depression was defined as CES-D-10 ≥8; (7) chronic kidney disease was defined as eGFR < 60 ml/min/1.73m2 or urinary albumin to creatinine ratio ≥3 mg/mmol.

**Figure 2:**
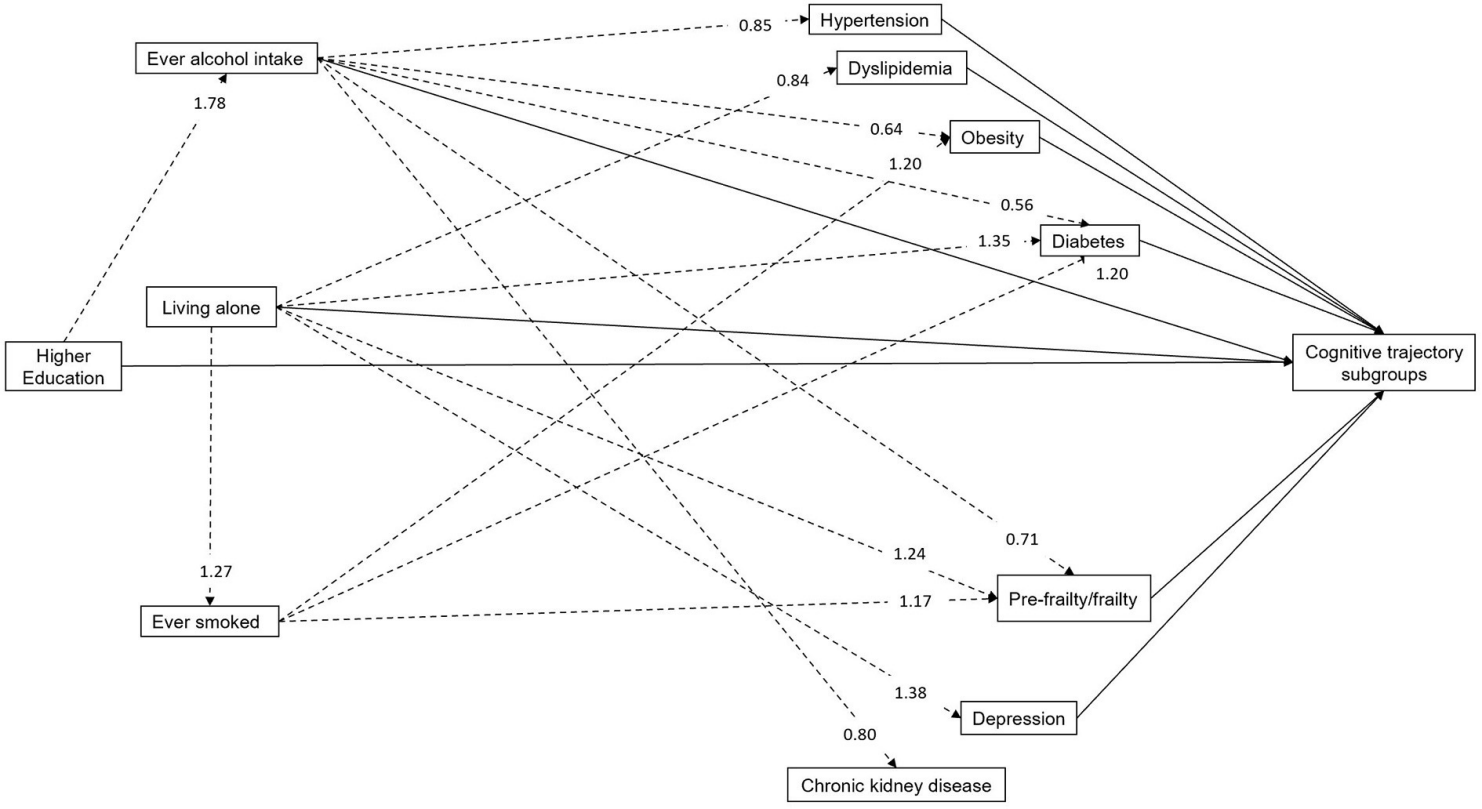
Relative risk ratios of path analysis for the indirect associations between modifiable factors and cognitive trajectory subgroups (*N* = 6,432). Solid arrows and dashed arrows refer to the indirect and direct associations with cognitive trajectory subgroups, respectively. The relative risk ratios of direct associations with cognitive trajectories are not shown here, but detailed in Table 2. Age, gender and ethnicity were also included in the full model.

Figure 2 shows the results of the path model (Akaike's Information Criteria: 86363.57; Bayesian Information Criteria: 87040.47), with the indirect associations between the social/behavioral factors and the selected chronic comorbidities. Education >12 years was associated with higher probability of being an ever alcohol drinker. Living alone was not only associated with an increased likelihood of smoking but increased the risk of diabetes, pre-frailty/frailty and depression. However, it was associated with a reduced risk of dyslipidemia. Similarly, being an ever smoker was associated with an elevated risk of diabetes, obesity and pre-frailty/frailty. In contrast, ever alcohol intake decreased the risk of most chronic comorbidities except dyslipidemia and depression. Full details of the indirect associations are shown in Supplementary Table 4.

Table 2 details the direct associations of the modifiable factors at baseline with cognitive trajectory subgroups from the SEM. There was no direct association between either smoking status or chronic kidney disease and cognitive trajectory subgroups, however all other factors predicted either the high-performer or the low-performer group, or both. In comparison with the average performers, >12 years of education, and ever alcohol intake predicted an increased likelihood of being a high performer and decreased risk of being a low performer. Conversely, pre-frailty/frailty predicted an increased risk of being in the low-performer group and a decreased likelihood of being in the high-performer group. However, six factors had unidirectional associations. Living alone and dyslipidemia each only predicted an increased risk of low cognitive performance but were not associated with less likelihood of being a high performer, while diabetes and depression, and in particular hypertension and obesity, predicted a decreased likelihood of being a high performer, but were not associated withlow cognitive performance. Findings were consistent when multinomial logistic regression was used instead of structural equation modeling.

**Table 2:** Direct association between modifiable factors and cognitive trajectory subgroups, with reference to average performers *(*N* = 6,432).

	**Highest performers**		**Lowest performer**	
	**(*****n*** = **2,298)**		**(*****n*** = **642)**	
	**Relative RR** **(95% CI)**	***P*-value**	**Relative RR** **(95% CI)**	***P*-value**
**Social/lifestyle factors**				
Education> 12 years	4.63 (4.07–5.29)	<0.001	0.71 (0.59–0.85)	<0.001
Living alone at home	0.89 (0.77–1.02)	0.09	1.26 (1.04–1.52)	0.02
Ever smoker	0.90 (0.79–1.03)	0.12	0.92 (0.76–1.11)	0.41
Ever alcohol intake	1.39 (1.17–1.66)	<0.001	0.79 (0.63–0.99)	0.04
**Chronic conditions**				
Hypertension	0.69 (0.60–0.80)	<0.001	0.89 (0.72–1.10)	0.28
Dyslipidemia	1.06 (0.93–1.22)	0.36	1.30 (1.07–1.57)	0.007
Obesity	0.84 (0.73–0.97)	0.01	0.89 (0.73–1.09)	0.26
Diabetes	0.69 (0.56–0.86)	0.001	1.27 (0.98–1.64)	0.07
Pre-frailty/frailty	0.60 (0.52–0.68)	<0.001	1.77 (1.47–2.14)	<0.001
Depression	0.68 (0.54–0.85)	0.001	1.23 (0.94–1.60)	0.13
Chronic kidney disease	0.87 (0.75–1.01)	0.06	1.00 (0.83–1.21)	0.99

RR, risk ratio. * Compared to average performers (n = 3,492), and additionally adjusted for age, gender and ethnicity. (1) hypertension was defined as on treatment for high BP or BP > 140/90 mmHg at study entry; (2) dyslipidemia was defined as those taking cholesterol-lowering medications or serum cholesterol ≥212 mg/dL (≥5 mmol/L; Australia) and ≥240 mg/dL (≥6.2 mmol/L; U.S.) or LDL > 160 mg/dL (>4.1 mmol/L); (3) obesity was defined as body mass index ≥30; (4) diabetes was defined from self-report or fasting glucose ≥126 mg/dL (≥7 mmol/L) or on treatment for diabetes; (5) frailty was defined using the adapted Fried frailty criteria, including being underweight, weak grip strength, exhaustion, slow walking speed and low physical activity, with pre-frail including anyone with 1 or 2 criteria and Frail as anyone with three or more criteria (22); (6) depression was defined as CES-D-10 ≥8; (7) chronic kidney disease was defined as eGFR < 60 ml/min/1.73 m2 or urinary albumin to creatinine ratio ≥3 mg/mmol.

In sensitivity analyses, the path analyses modeling high and low cognitive performance separately showed no material difference in any of the direct or indirect associations (Supplementary Tables 5–7). The multinomial logistic regression produced largely consistent results in terms of the direct associations, although associations were slightly attenuated in subgroup analyses (Supplementary Tables 8–10). When all seven trajectory classes were included, very similar patterns of association were observed (Supplementary Tables 11, 12).

## Discussion

This is the first study to investigate the modifiable factors for high and low cognitive performance separately, as well as the potential mediating roles of a number of behavioral factors and chronic conditions in relation to cognitive function. Compared with average performers, a number of modifiable factors directly predicted both high cognitive performance, and in the reverse direction, were protective against low cognitive performance (the overall summary of findings is shown in Supplementary Figure 2). The former included high education and alcohol intake, as well as the absence of pre-frailty/frailty. However, some other factors were specifically associated with one group only. The absence of hypertension and obesity predicted high cognitive performance only, while living alone and dyslipidemia only predicted low cognitive performance. This suggests that more targeted approaches may be needed to promote high cognitive function as distinct from delaying cognitive decline. In addition, we also found that a number of chronic comorbidities might be plausible intermediates of the association between behavioral factors and cognitive function. These indicate that behavior modification, social support and prevention of these chronic comorbidities may be suitable targets to protect late-life cognitive function.

Most chronic comorbidities in our framework were associated with lower cognitive performance, consolidating the existing evidence of the importance of cardiometabolic health, non-frailty, renal function and mental health for cognitive function (2, 13). The four cardiometabolic conditions–hypertension, diabetes, obesity and dyslipidemia–are well-known vascular risk factors of Alzheimer's disease and cerebrovascular damage and often co-exist (28). It is worth noting that dyslipidemia predicted low cognitive performance in this study, although evidence about the association between late-life serum cholesterol and the risk of dementia is relatively weak (29). Our findings add to the current research that healthy blood lipids may not additively benefit cognitive aging, but once they become abnormal (serum cholesterol ≥ 212 mg/dL or low-density lipoproteins > 160 mg/dL), may still negatively affect cognitive function of older adults, even though may not lead to a dementia diagnosis clinically. Other chronic comorbidities might also interact with these cardiometabolic conditions. For example, depression might increase the risk of vascular diseases, in addition to its direct effects on cognitive function *via* neuroinflammation and hippocampal atrophy (30). Similarly, these cardiometabolic conditions are also common in frail older adults and patients with chronic kidney disease (31, 32).

Our study found that obesity and hypertension were associated with a decreased likelihood of high cognitive performance compared with stable performance, but neither was associated with low cognitive performance. Although evidence for mid-life obesity and hypertension as risk factors for cognitive decline is strong (2, 33), the influence of these two conditions in late life remains unclear (8, 34). The mixed results from previous studies might be partially explained by reverse causation. Weight loss is not uncommon in the early stages of dementia (35) and frailty with malnutrition might precede cognitive impairment (36). Indeed, some studies observed that weight loss and being underweight were associated with cognitive decline in older adults (37, 38). Blood pressure generally tends to increase with age (39), which might help prevent low cerebral perfusion and therefore protect neurons at old age (40). However, recent evidence indicates that decreasing patterns of blood pressure from mid-life to late life increased the risk of cognitive decline (33). Despite being less understood, hypotension may be a greater risk factor for cognitive decline, due to impaired autoregulation (34).

However, despite obesity and hypertension not increasing the risk of low cognitive performance among older adults in this study, they were still negatively associated with high cognitive performance. Obesity is a recognized risk factor for many conditions with negative effects on late-life cognition, such as diabetes and chronic kidney disease (41, 42), and may itself be amenable to dietary and exercise intervention. Similarly, sustained hypertension elevates the risk of various cerebrovascular diseases which affect cognitive function (33), and controlling blood pressure at a normal level in late life is protective (43). Our results should be interpreted with caution since the life-course patterns of BMI and blood pressure were not available. However, given the results from this study, control of body weight and blood pressure (44), are important goals to maximize the cognitive function of older adults.

As hypothesized in our theoretical framework, three behavioral factors examined-living status, alcohol intake and smoking-were associated with the risk of several chronic comorbidities examined, and both living status and alcohol intake also directly predicted cognitive function. Despite smoking being linked to inflammation and oxidative stress (45), no direct effect on cognition was observed in this study. This is in contrast to some previous research (46), although these prior studies have not attempted to distinguish direct and indirect associations. Smoking was associated with an increased risk of obesity, diabetes and frailty in our study, all of which in turn, were associated with cognitive function. Therefore, smoking cessation should still be a target for dementia prevention. As for alcohol intake, beneficial effects were shown in better cognitive function and lower risk of multiple comorbidities. The benefits of low-to-moderate alcohol intake to cognitive function was observed in previous research (47). However, excess alcohol intake has been shown to be adverse to cognitive function (2), so alcohol intake might be protective only at a low-to-moderate level.

The strengths of this study include the large sample size, longitudinal assessment of cognitive function, with the majority of participants having three or more repeated cognitive assessments (Supplementary Table 13), and the analysis of the direct/indirect associations of a number of modifiable factors with cognitive function. Also, we investigated the predictors of high and low cognitive performance separately, providing information for more precise interventions. More importantly, we employed a group-based method that is data-driven and hypothesis-free to identify inter-individual variability in cognitive performance. Previous research has often partitioned the population based on subjective classification criteria (e.g., <1 standard deviation cognitive changes) (4), making assumptions about the degree of population heterogeneity a priori. Yet, there is currently no well-established clinical threshold for any of the four cognitive tests. Therefore, our joint modeling approach enables the identification of a subgroup with the highest performance across all cognitive tests, which aligns with the aim of our study, to identify potential modifiable factors associated with not only low cognitive trajectories but high cognitive performer. However, there are several limitations. First, the ASPREE study is comprised of a group of older adults who were generally healthy at baseline. Therefore, the results are not necessarily generalizable to all community-dwelling older individuals and are likely to oversample higher cognitive performance and underestimate the prevalence of chronic conditions. However, the majority of the participants were enrolled through partnership with primary care providers across a wide range of areas, with varying socioeconomic and health status. For example, 29% of the participants had obesity, and 75% had hypertension at enrolment. This indicates that the study sample is broadly representative of the general older population. Second, the SEM was restricted to a subsample, meaning the findings may be only applicable to individuals following particular cognitive trajectories. Nevertheless, these results remained largely unchanged in sensitivity analyses involving the entire sample across the seven subgroups. Third, we did not have data on physical activity and dietary patterns for the entire cohort, which are two established risk factors for cognitive function (2). Nevertheless, these were likely to be partly reflected by obesity in the analysis. Fourth, to facilitate comprehensive multivariable analysis but avoid excessive path parameters in the model, all “exposure” variables were dichotomized. This approach, however, does limit the ability to investigate non-binary associations (48, 49). Fifth, the theoretical framework simulates only one of the many scenarios and all variables except for the cognitive trajectories, were assessed cross-sectionally. The arrows between behavioral factors and chronic conditions can be bidirectional with mutual effects (e.g., smoking and depression), and possibly synergistic and multiplicative.

## Conclusion

Maintaining healthy body weight, normal blood pressure and blood glucose, as well as prevention and treatment of depression may help maintain high cognitive function for older adults. Prevention of dyslipidemia may be protective against low cognitive performance and cognitive decline in late life. Frailty prevention should also be advocated for strategies of healthy cognitive aging. Smoking cessation, low-to-moderate alcohol intake and proactive support for those living alone may be beneficial for older adults, given the potential direct and indirect effects these factors have on cognitive function.

## Data availability statement

The data used in this study is available for access, following formal approval through an application process. Further inquiries can be directed to the corresponding author (joanne.ryan@monash.edu) and the ASPREE investigation team (https://aspree.org/).

## Ethics statement

The ASPREE clinical trial was conducted according to the guidelines of the International Conference on Harmonization Good Clinical Practice. The participants provided written informed consent to participate in the trial. The current study was approved by the Monash University Human Research Ethics Committee.

## Author contributions

Study concept and design: JR, ZW, RLW, and TC. Trial design and Acquisition of data: JM, AM, RLW, RS, RW, ES, and SO. Analysis of data: ZW. Drafting of the manuscript: ZW and JR. Approval of the final version to be submitted, critically revising and editing the manuscript, and interpretation of data: All authors.

## Funding

This work was supported by the National Institute on Aging and the National Cancer Institute at the National Institutes of Health (Grant Numbers: U01AG029824 and U19AG062682); and the National Health and Medical Research Council (NHMRC) of Australia (Grant Numbers: 334047 and 1127060); and Monash University (Australia); and the Victorian Cancer Agency (Australia). JR was supported by an NHMRC Dementia Research Leader Fellowship (Grant Number: APP1135727). ZW is a recipient of the RTP scholarship awarded by Monash University and the Australian Government. The funders had no role in study design and collection, analysis, and interpretation of the results.

## Conflict of interest

Author AM reports receiving consulting fees from Alkahest, Inc. and grants from the National Institute on Aging. RS reports grants for clinical research regarding dementia and Alzheimer's disease from the National Institutes of Health, the Centers for Medicare and Medicaid Services, the Department of Defense, and the Illinois Department of Public Health; being as a non-compensated member of the Board of Directors of the Alzheimer's Association–Illinois Chapter; and being as a site principal investigator or sub-investigator for clinical trials and research studies for which his institution (Rush University Medical Center) is sponsored (Amylyx Pharmaceuticals, Inc., Eli Lilly and Co., Inc., Genentech, Inc., Lundbeck, Inc., Merck and Co., Inc., Navidea Biopharmaceuticals, Novartis Pharmaceuticals, Inc., Roche Holdings AG, and Takeda Development Center Americas, Inc.).

The remaining authors declare that the research was conducted in the absence of any commercial or financial relationships that could be construed as a potential conflict of interest.

## Publisher's note

All claims expressed in this article are solely those of the authors and do not necessarily represent those of their affiliated organizations, or those of the publisher, the editors and the reviewers. Any product that may be evaluated in this article, or claim that may be made by its manufacturer, is not guaranteed or endorsed by the publisher.

## References

[1] Harada CN Natelson Love MC Triebel KL. Normal cognitive aging. Clin Geriatr Med. (2013) 29:737–52. 10.1016/j.cger.2013.07.002 24094294 PMC4015335

[2] Livingston G Huntley J Sommerlad A Ames D Ballard C Banerjee S. Dementia prevention, intervention, and care: 2020 report of the lancet commission. Lancet. (2020) 396:413–46. 10.1016/S0140-6736(20)30367-6 32738937 PMC7392084

[3] Daffner KR. Promoting successful cognitive aging: a comprehensive review. J Alzheimers Dis. (2010) 19:1101–22. 10.3233/JAD-2010-1306 20308777 PMC3047597

[4] Wu Z Phyo AZZ Al-Harbi T Woods RL Ryan J. Distinct cognitive trajectories in late life and associated predictors and outcomes: a systematic review. J Alzheimers Dis Rep. (2020) 4:459–78. 10.3233/ADR-200232 33283167 PMC7683100

[5] Wu Z Woods RL Wolfe R Storey E Chong TTJ Shah RC. Trajectories of cognitive function in community-dwelling older adults: a longitudinal study of population heterogeneity. Alzheimers Dement. (2021) 13:e12180. 10.1002/dad2.12180 33969173 PMC8088593

[6] McFall GP McDermott KL Dixon RA. Modifiable risk factors discriminate memory rrajectories in non-demented aging: precision factors and targets for promoting healthier brain aging and preventing dementia. J Alzheimers Dis. (2019) 70:S101–S18. 10.3233/JAD-180571 30775975 PMC6700610

[7] Howrey BT Raji MA Masel MM Peek MK. Stability in cognitive function over 18 years: prevalence and predictors among older Mexican Americans. Curr Alzheimer Res. (2015) 12:614–21. 10.2174/1567205012666150701102947 26239038 PMC5501462

[8] Pedditzi E Peters R Beckett N. The risk of overweight/obesity in mid-life and late life for the development of dementia: a systematic review and meta-analysis of longitudinal studies. Age Ageing. (2016) 45:14–21. 10.1093/ageing/afv151 26764391

[9] Gifford KA Badaracco M Liu D Tripodis Y Gentile A Lu Z. Blood pressure and cognition among older adults: a meta-analysis. Arch Clin Neuropsychol. (2013) 28:649–64. 10.1093/arclin/act046 23838685 PMC3807830

[10] Xu W Wang H Wan Y Tan C Li J Tan L. Alcohol consumption and dementia risk: a dose-response meta-analysis of prospective studies. Eur J Epidemiol. (2017) 32:31–42. 10.1007/s10654-017-0225-3 28097521

[11] Beydoun MA Beydoun HA Gamaldo AA Teel A Zonderman AB Wang Y. Epidemiologic studies of modifiable factors associated with cognition and dementia: systematic review and meta-analysis. BMC Public Health. (2014) 14:643. 10.1186/1471-2458-14-643 24962204 PMC4099157

[12] Brunello G Fort M Schneeweis N Winter-Ebmer R. The causal effect of education on health: what is the role of health behaviors? Health Econ. (2016) 25:314–36. 10.1002/hec.3141 25581162

[13] Etgen T Chonchol M Forstl H Sander D. Chronic kidney disease and cognitive impairment: a systematic review and meta-analysis. Am J Nephrol. (2012) 35:474–82. 10.1159/000338135 22555151

[14] Sommerlad A Ruegger J Singh-Manoux A Lewis G Livingston G. Marriage and risk of dementia: systematic review and meta-analysis of observational studies. J Neurol Neurosurg Psychiatry. (2018) 89:231–8. 10.1136/jnnp-2017-316274 29183957 PMC5869449

[15] Aspree Investigator Group. Study design of ASPirin in Reducing Events in the Elderly (ASPREE): a randomized, controlled trial. Contemp Clin Trials. (2013) 36:555–64. 10.1016/j.cct.2013.09.014 24113028 PMC3919683

[16] Ryan J Storey E Murray AM Woods RL Wolfe R Reid CM. Randomized placebo-controlled trial of the effects of aspirin on dementia and cognitive decline. Neurology. (2020) 95:e320–e31. 10.1212/WNL.0000000000009277 32213642 PMC7455352

[17] Teng EL Chui HC. The Modified Mini-Mental State (3MS) examination. J Clin Psychiatry. (1987) 48:314–8. 3611032

[18] Ross TP. The reliability of cluster and switch scores for the controlled oral word association test. Arch Clin Neuropsychol. (2003) 18:153–64. 10.1093/arclin/18.2.153 14591467

[19] Ryan J Woods RL Murray AM Shah RC Britt CJ Reid CM. Normative performance of older individuals on the Hopkins Verbal Learning Test-Revised (HVLT-R) according to ethno-racial group, gender, age and education level. Clin Neuropsychol. (2020) 35:1174–90. 10.1080/13854046.2020.1730444 32100619 PMC7483610

[20] Sheridan LK Fitzgerald HE Adams KM Nigg JT Martel MM Puttler LI. Normative symbol digit modalities test performance in a community-based sample. Arch Clin Neuropsychol. (2006) 21:23–8. 10.1016/j.acn.2005.07.003 16139470

[21] Irwin M Artin KH Oxman MN. Screening for depression in the older adult: criterion validity of the 10-item Center for Epidemiological Studies Depression Scale (CES-D). Arch Intern Med. (1999) 159:1701–4. 10.1001/archinte.159.15.1701 10448771

[22] Fried LP Tangen CM Walston J Newman AB Hirsch C Gottdiener J. Frailty in older adults: evidence for a phenotype. J Gerontol A Biol Sci Med Sci. (2001) 56:M146–56. 10.1093/gerona/56.3.M146 11253156

[23] Wolfe R Murray AM Woods RL Kirpach B Gilbertson D Shah RC. The aspirin in reducing events in the elderly trial: statistical analysis plan. Int J Stroke. (2018) 13:335–8. 10.1177/1747493017741383 29111960 PMC6380180

[24] Nagin DS. Group-based trajectory modeling: an overview. Ann Nutr Metab. (2014) 65:205–10. 10.1159/000360229 25413659

[25] Nagin DS Jones BL Passos VL Tremblay RE. Group-based multi-trajectory modeling. Stat Methods Med Res. (2018) 27:2015–23. 10.1177/0962280216673085 29846144

[26] Streiner DL. Finding our way: an introduction to path analysis. Can J Psychiatry. (2005) 50:115–22. 10.1177/070674370505000207 15807228

[27] McNeil JJ Woods RL Nelson MR Murray AM Reid CM Kirpach B. Baseline characteristics of participants in the ASPREE (ASPirin in Reducing Events in the Elderly) study. J Gerontol A Biol Sci Med Sci. (2017) 72:1586–93. 10.1093/gerona/glw342 28329340 PMC5861878

[28] Takeda S Rakugi H Morishita R. Roles of vascular risk factors in the pathogenesis of dementia. Hypertens Res. (2020) 43:162–7. 10.1038/s41440-019-0357-9 31723253

[29] Reitz C. Dyslipidemia and dementia: current epidemiology, genetic evidence, and mechanisms behind the associations. J Alzheimers Dis. (2012) 30:S127–45. 10.3233/JAD-2011-110599 21965313 PMC3689537

[30] Byers AL Yaffe K. Depression and risk of developing dementia. Nat Rev Neurol. (2011) 7:323–31. 10.1038/nrneurol.2011.60 21537355 PMC3327554

[31] Grande G Haaksma ML Rizzuto D Melis RJF Marengoni A Onder G. Co-occurrence of cognitive impairment and physical frailty, and incidence of dementia: Systematic review and meta-analysis. Neurosci Biobehav Rev. (2019) 107:96–103. 10.1016/j.neubiorev.2019.09.001 31491474

[32] Gansevoort RT Correa-Rotter R Hemmelgarn BR Jafar TH Heerspink HJ Mann JF. Chronic kidney disease and cardiovascular risk: epidemiology, mechanisms, and prevention. Lancet. (2013) 382:339–52. 10.1016/S0140-6736(13)60595-4 23727170

[33] Walker KA Sharrett AR Wu A Schneider ALC Albert M Lutsey PL. Association of midlife to late-life blood pressure patterns with incident dementia. JAMA. (2019) 322:535–45. 10.1001/jama.2019.10575 31408138 PMC6692677

[34] Ou YN Tan CC Shen XN Xu W Hou XH Dong Q. Blood pressure and risks of cognitive impairment and dementia: a systematic review and meta-analysis of 209 prospective studies. Hypertension. (2020) 76:217–25. 10.1161/HYPERTENSIONAHA.120.14993 32450739

[35] Sobow T Fendler W Magierski R. Body mass index and mild cognitive impairment-to-dementia progression in 24 months: a prospective study. Eur J Clin Nutr. (2014) 68:1216–9. 10.1038/ejcn.2014.167 25117990

[36] Bell SP Liu D Samuels LR Shah AS Gifford KA Hohman TJ. Late-life body mass index, rapid weight loss, apolipoprotein E epsilon4 and the risk of cognitive decline and incident dementia. J Nutr Health Aging. (2017) 21:1259–67. 10.1007/s12603-017-0906-3 29188888 PMC5736008

[37] Singh-Manoux A Dugravot A Shipley M Brunner EJ Elbaz A Sabia S. Obesity trajectories and risk of dementia: 28 years of follow-up in the Whitehall II Study. Alzheimers Dement. (2018) 14:178–86. 10.1016/j.jalz.2017.06.2637 28943197 PMC5805839

[38] Qu Y Hu HY Ou YN Shen XN Xu W Wang ZT. Association of body mass index with risk of cognitive impairment and dementia: a systematic review and meta-analysis of prospective studies. Neurosci Biobehav Rev. (2020) 115:189–98. 10.1016/j.neubiorev.2020.05.012 32479774

[39] Li W He Y Xia L Yang X Liu F Ma J. Association of age-related trends in blood pressure and body composition indices in healthy adults. Front Physiol. (2018) 9:1574. 10.3389/fphys.2018.01574 30534075 PMC6275465

[40] Yang YH Roe CM Morris JC. Relationship between late-life hypertension, blood pressure, and Alzheimer's disease. Am J Alzheimers Dis Other Demen. (2011) 26:457–62. 10.1177/1533317511421779 21921085 PMC3312309

[41] Bellou V Belbasis L Tzoulaki I Evangelou E. Risk factors for type 2 diabetes mellitus: An exposure-wide umbrella review of meta-analyses. PLoS ONE. (2018) 13:e0194127. 10.1371/journal.pone.0194127 29558518 PMC5860745

[42] Kazancioglu R. Risk factors for chronic kidney disease: an update. Kidney *Int Suppl (2011)*. (2013) 3:368–71. 10.1038/kisup.2013.79 25019021 PMC4089662

[43] Research Group SPRINT Williamson JD Pajewski NM Auchus AP Bryan RN Chelune G. Effect of intensive vs standard blood pressure control on probable dementia: a randomized clinical trial. JAMA. (2019) 321:553–61. 10.1001/jama.2018.21442 30688979 PMC6439590

[44] Rouch L Cestac P Hanon O Cool C Helmer C Bouhanick B. Antihypertensive drugs, prevention of cognitive decline and dementia: a systematic review of observational studies, randomized controlled trials and meta-analyses, with discussion of potential mechanisms. CNS Drugs. (2015) 29:113–30. 10.1007/s40263-015-0230-6 25700645

[45] Bruno RS Traber MG. Vitamin E biokinetics, oxidative stress and cigarette smoking. Pathophysiology. (2006) 13:143–9. 10.1016/j.pathophys.2006.05.003 16814530

[46] Zhong G Wang Y Zhang Y Guo JJ Zhao Y. Smoking is associated with an increased risk of dementia: a meta-analysis of prospective cohort studies with investigation of potential effect modifiers. PLoS ONE. (2015) 10:e0118333. 10.1371/journal.pone.0118333 25763939 PMC4357455

[47] Rehm J Hasan OSM Black SE Shield KD Schwarzinger M. Alcohol use and dementia: a systematic scoping review. Alzheimers Res Ther. (2019) 11:1. 10.1186/s13195-018-0453-0 30611304 PMC6320619

[48] Aune D Sen A Prasad M Norat T Janszky I Tonstad S. BMI and all cause mortality: systematic review and non-linear dose-response meta-analysis of 230 cohort studies with 3. 74 million deaths among 303 million participants. BMJ. (2016) 353:i2156. 10.1136/bmj.i2156 27146380 PMC4856854

[49] Brunstrom M Carlberg B. Association of blood pressure lowering with mortality and cardiovascular disease across blood pressure levels: a systematic review and meta-analysis. JAMA Intern Med. (2018) 178:28–36. 10.1001/jamainternmed.2017.6015 29131895 PMC5833509

