# Supplementary material for: Potential modifiable factors associated with late-life cognitive trajectories
Source: Front Neurol. 2022 Aug 3;13:950644. doi: 10.3389/fneur.2022.950644 (PMC9381981; doi:10.3389/fneur.2022.950644)
Supplement: Supplementary file 1 [file Data_Sheet_1.docx]

**Supplementary materials**

**Inclusion and exclusion criteria of the ASPREE clinical trial (1)**

**Inclusion criteria:**

1) non-minority men and women 70 years of age and older;

2) US minority (African American and Hispanic) 65 years of age and older;

3) Willing and able to provide informed consent.

**Exclusion criteria:**

1) a history of a diagnosed cardiovascular event defined as myocardial infarction, heart failure, angina pectoris, stroke, transient ischemic attack, >50% carotid stenosis or previous carotid endarterectomy or stenting, coronary artery angioplasty or stenting, coronary artery bypass grafting, or abdominal aortic aneurysm;

2) a clinical diagnosis of atrial fibrillation;

3) serious illness likely to cause death within the next 5 years;

4) a current or recurrent condition with a high risk of major bleeding;

5) anemia (hemoglobin <12 g/dl males, <11 g/dl females);

6) an absolute contraindication or allergy to aspirin;

7) current participation in an ongoing clinical trial;

8) current use of aspirin for secondary prevention;

9) current continuous use of other antiplatelet drug or anticoagulant;

10) a systolic blood pressure ≥180 mm Hg and/or a diastolic blood pressure ≥105 mm Hg;

11) a history of dementia or a Modified Mini-Mental State Examination (3MS) score ≤77;

12) severe difficulty or an inability to perform any one of the 6 Katz activities of daily living;

13) pill-taking compliance <80% during a 4-week placebo run-in phase.

**Supplementary Table 1. Comparison between participants excluded and included in the trajectory modeling (N=19,114)**

|  | **Included (17,724, 92.7%)** | **Excluded**  **(1,390, 7.3%)** | **P-value** |
| --- | --- | --- | --- |
| Age, years |  |  | <0.001 |
| 65-69 * | 483 (2.7) | 80 (5.8) |  |
| 65-74 | 9,945 (56.1) | 656 (47.2) |  |
| 75-84 | 6,658 (37.6) | 560 (40.3) |  |
| ≥85 | 638 (3.6) | 94 (6.8) |  |
| Gender |  |  | 0.03 |
| Men | 7,764 (43.8) | 568 (40.9) |  |
| Women | 9,960 (56.2) | 822 (59.1) |  |
| Ethnicity |  |  | <0.001 |
| AU White | 15,249 (86.0) | 1,113 (80.1) |  |
| US White | 1,020 (5.8) | 68 (4.9) |  |
| African American | 765 (4.3) | 136 (9.8) |  |
| Hispanic/Latino | 434 (2.5) | 54 (3.9) |  |
| Others | 256 (1.4) | 19 (1.4) |  |
| Education, years |  |  | 0.05 |
| ≤ 12 | 7,974 (45.0) | 662 (47.7) |  |
| > 12 | 9,750 (55.0) | 727 (52.3) |  |
| Living alone at home |  |  | <0.001 |
| Yes | 5,732 (32.3) | 519 (37.3) |  |
| No | 11,992 (67.7) | 871 (62.7) |  |
| Ever smoker |  |  | <0.001 |
| Yes | 7,835 (44.2) | 699 (50.3) |  |
| No | 9,889 (55.8) | 691 (49.7) |  |
| Ever alcohol intake |  |  | <0.001 |
| Yes | 14,689 (82.9) | 1,089 (78.4) |  |
| No | 3,035 (17.1) | 301 (21.6) |  |
| Hypertension |  |  | 0.01 |
| Yes | 13,123 (74.0) | 1,072 (77.1) |  |
| No | 4,601 (26.0) | 318 (22.9) |  |
| Dyslipidemia |  |  | 0.15 |
| Yes | 11,585 (65.4) | 882 (63.5) |  |
| No | 6,139 (34.6) | 508 (36.5) |  |
| Obesity |  |  | 0.96 |
| Yes | 5,203 (29.5) | 406 (29.6) |  |
| No | 12,448 (70.5) | 968 (70.4) |  |
| Diabetes |  |  | 0.01 |
| Yes | 1,869 (10.5) | 176 (12.7) |  |
| No | 15,855 (89.5) | 1,214 (87.3) |  |
| Pre-frailty/frailty |  |  | <0.001 |
| Yes | 7,134 (40.3) | 734 (52.8) |  |
| No | 10,590 (59.7) | 656 (47.2) |  |
| Depression |  |  | <0.001 |
| Yes | 1,702 (9.6) | 177 (12.7) |  |
| No | 16,019 (90.4) | 1,212 (87.3) |  |
| Chronic Kidney disease |  |  | <0.001 |
| Yes | 4,331 (26.2) | 409 (32.3) |  |
| No | 12,166 (73.8) | 858 (67.7) |  |

* Only includes U.S. African American or Hispanic/Latino participants, who were eligible to enroll from 65 years or above (all other participants needed to be 70 years or above to be recruited).

Note: 1) hypertension was defined as on treatment for high BP or BP >140/90 mmHg at study entry; 2) dyslipidemia was defined as those taking cholesterol-lowering medications or serum cholesterol ≥212 mg/dL (≥5 mmol/L; Australia) and ≥240 mg/dL (≥6.2 mmol/L; U.S.) or LDL > 160 mg/dL (>4.1 mmol/L); 3) obesity was defined as body mass index ≥30; 4) diabetes was defined from self-report or fasting glucose ≥126mg/dL (≥7 mmol/L) or on treatment for diabetes; 5) frailty was defined using the adapted Fried frailty criteria, including being underweight, weak grip strength, exhaustion, slow walking speed and low physical activity, with pre-frail including anyone with 1 or 2 criteria and Frail as anyone with three or more criteria (2); 6) depression was defined as CES-D-10 ≥8; 7) chronic kidney disease was defined as eGFR < 60 ml/min/1.73m2 or urinary albumin to creatinine ratio ≥3 mg/mmol.

**Supplementary Table 2. Process of model selection and assessment of adequacy and fit of the selected trajectory model (N=17,724)**

|  | **AIC** | **BIC** | **Adjusted BIC** | |
| --- | --- | --- | --- | --- |
| **Model fit** |  |  |  | |
| 1-class model | -727283.38 | -727345.69 | -727330.07 | |
| 2-class model | -695084.86 | -695193.92 | -695166.58 | |
| 3-class model | -683791.90 | -683947.69 | -683908.64 | |
| 4-class model | -678914.08 | -679116.61 | -679065.84 | |
| 5-class model | -674241.52 | -674490.78 | -674428.30 | |
| 6-class model | -671733.85 | -672029.86 | -671955.66 | |
| 7-class model ** | -669372.20 | -669714.94 | -669629.03 | |
| 8-class model * | -667217.17 | -667606.65 | -667509.02 | |
|  |  | | | |
|  | **N (%)** | **AvePP (%)** | **OCC** | **EP (%)** |
| **Model adequacy** |  |  |  |  |
| Total | 17,724 |  |  |  |
| Class 1 | 2,512 (14.2) | 89.8 | 57.7 | 13.2 |
| Class 2 | 3,072 (17.3) | 85.0 | 28.9 | 16.4 |
| Class 3 | 3,526 (19.9) | 84.1 | 23.7 | 18.2 |
| Class 4 | 3,835 (21.6) | 85.6 | 24.3 | 19.7 |
| Class 5 | 2,063 (11.6) | 84.2 | 43.0 | 11.0 |
| Class 6 | 1,996 (11.3) | 88.1 | 63.4 | 10.4 |
| Class 7 | 720 (4.1) | 93.3 | 352.0 | 3.8 |

AvePP, average posterior probability; OCC, odds of correct classification; AIC, Akaike information criterion; BIC, Bayesian information criterion; EP, estimated probability; ** The model was selected; * The model was rejected based on one or more model selection criteria

Note: 1) group-based multi-trajectory modeling only included those with available data of all the four cognitive tests at baseline and at least one subsequent timepoint, thus a number of 17,724 participants were eligible; 2) model selection criteria: a. model fit (assessed by Akaike information criterion, Bayesian information criterion, and adjusted Bayesian information criterion); b. average posterior probability >0.7 for all classes; c. odds of correct classification >5 for all classes; d. a reasonably narrow confidence interval for each trajectory; e. close correspondence between estimated probabilities and the percentage of participants assigned to a class; f. a trajectory with the highest baseline score and the lowest rate of decline across all cognitive tests, as this would help address of our aim of investigating high cognitive performance.

**Supplementary Table 3. Intercept and slope parameters of the identified latent classes (N=17,724)**

|  | **Class 1** | **Class 2** | **Class 3** | **Class 4** | **Class 5** | **Class 6** | **Class 7** |
| --- | --- | --- | --- | --- | --- | --- | --- |
| **3MS** |  |  |  |  |  |  |  |
| Intercept | 97.52 | 96.71 | 94.92 | 94.20 | 91.20 | 89.75 | 85.50 |
| Slope | 0.13 | 0.11 | 0.12 | 0.03 | -0.20 | -0.46 | -2.75 |
| **COWAT** |  |  |  |  |  |  |  |
| Intercept | 16.09 | 14.22 | 12.56 | 11.48 | 11.56 | 9.55 | 9.57 |
| Slope | 0.39 | 0.29 | 0.23 | 0.15 | 0.12 | 0.04 | -0.17 |
| **HVLT-R** |  |  |  |  |  |  |  |
| Intercept | 11.01 | 10.83 | 7.73 | 8.20 | 5.19 | 5.76 | 3.66 |
| Slope | 0.23 | 0.20 | 0.09 | 0.05 | -0.19 | -0.25 | -0.75 |
| **SDMT** |  |  |  |  |  |  |  |
| Intercept | 49.62 | 38.35 | 44.02 | 30.75 | 36.79 | 24.65 | 25.57 |
| Slope | -0.34 | -0.48 | -0.52 | -0.52 | -0.70 | -0.62 | -1.18 |

3MS, Modified Mini-Mental State Examination; COWAT-F, Controlled Oral Word Association Test-F; HVLT-R, Hopkins Verbal Learning Test–Revised (delayed recall); SDMT, Symbol Digit Modalities Test

Note: intercept parameters refer to the raw scores at baseline, and slope parameters refer to rate of change per year.

**Supplementary Table 4. Associations between education and behaviors, and associated factors, including chronic conditions (N=6,432)**

|  | **Odds ratio (95% CI)** | **P-value** |
| --- | --- | --- |
| Education> 12 years (versus education≤ 12 years) -> |  |  |
| Living alone at home | 0.95 (0.85-1.06) | 0.38 |
| Ever smoker | 1.05 (0.95-1.17) | 0.33 |
| Ever alcohol intake | 1.78 (1.55-2.05) | <0.001 |
| Living alone at home (versus living with someone else) -> |  |  |
| Ever smoker | 1.27 (1.14-1.43) | <0.001 |
| Ever alcohol intake | 1.07 (0.93-1.24) | 0.33 |
| Hypertension | 0.96 (0.85-1.09) | 0.57 |
| Dyslipidemia | 0.84 (0.75-0.95) | 0.005 |
| Diabetes | 1.35 (1.14-1.62) | 0.001 |
| Obesity | 1.08 (0.96-1.22) | 0.20 |
| Pre-frailty/frailty | 1.24 (1.11-1.39) | <0.001 |
| Depression | 1.38 (1.16-1.66) | <0.001 |
| Chronic kidney disease | 1.10 (0.97-1.25) | 0.12 |
| Ever smoker (versus never smoked) -> |  |  |
| Hypertension | 1.10 (0.98-1.24) | 0.12 |
| Dyslipidemia | 1.11 (0.99-1.24) | 0.08 |
| Diabetes | 1.20 (1.01-1.43) | 0.04 |
| Obesity | 1.20 (1.07-1.34) | 0.002 |
| Pre-frailty/frailty | 1.17 (1.05-1.31) | 0.005 |
| Depression | 1.13 (0.94-1.35) | 0.20 |
| Chronic kidney disease | 1.10 (0.98-1.24) | 0.12 |
| Ever alcohol intake (versus never drunk alcohol) -> |  |  |
| Hypertension | 0.85 (0.72-0.99) | 0.04 |
| Dyslipidemia | 1.10 (0.95-1.28) | 0.22 |
| Diabetes | 0.56 (0.46-0.69) | <0.001 |
| Obesity | 0.64 (0.55-0.74) | <0.001 |
| Pre-frailty/frailty | 0.71 (0.61-0.82) | <0.001 |
| Depression | 0.93 (0.74-1.17) | 0.55 |
| Chronic kidney disease | 0.80 (0.68-0.93) | 0.004 |

Note: 1) age, gender and ethnicity were also included in the full model; 2) variables with arrows behind are independent variables, and those below the independent variables are dependent variables; 3) hypertension was defined as on treatment for high BP or BP >140/90 mmHg at study entry; 4) Dyslipidemia was defined as those taking cholesterol-lowering medications or serum cholesterol ≥212 mg/dL (≥5 mmol/L; Australia) and ≥240 mg/dL (≥6.2 mmol/L; U.S.) or LDL > 160 mg/dL (>4.1 mmol/L); 5) obesity was defined as body mass index ≥30; 6) diabetes was defined from self-report or fasting glucose ≥126mg/dL (≥7 mmol/L) or on treatment for diabetes; 7) frailty was defined using the adapted Fried frailty criteria, including being underweight, weak grip strength, exhaustion, slow walking speed and low physical activity, with pre-frail including anyone with 1 or 2 criteria and Frail as anyone with three or more criteria (2); 8) depression was defined as CES-D-10 ≥8; 9) chronic kidney disease was defined as eGFR < 60 ml/min/1.73m2 or urinary albumin to creatinine ratio ≥3 mg/mmol.

**Supplementary Table 5. Direct association between modifiable factors and cognitive trajectory subgroups, with reference to average performers ^a^**

|  | **Highest performers**  **(n=2,298) ^b^** | | **Lowest performer**  **(n=642) ^c^** | |
| --- | --- | --- | --- | --- |
|  | **Relative RR**  **(95% CI)** | **P-value** | **Relative RR**  **(95% CI)** | **P-value** |
| **Social/lifestyle factors** |  |  |  |  |
| Education> 12 years | 4.72 (4.13-5.39) | <0.001 | 0.71 (0.59-0.85) | <0.001 |
| Living alone at home | 0.87 (0.76-1.00) | 0.05 | 1.24 (1.02-1.50) | 0.03 |
| Ever smoker | 0.91 (0.80-1.03) | 0.13 | 0.91 (0.76-1.10) | 0.34 |
| Ever alcohol intake | 1.38 (1.15-1.64) | <0.001 | 0.78 (0.62-0.98) | 0.04 |
| **Chronic conditions** |  |  |  |  |
| Hypertension | 0.69 (0.60-0.79) | <0.001 | 0.89 (0.72-1.10) | 0.28 |
| Dyslipidemia | 1.08 (0.94-1.23) | 0.26 | 1.30 (1.07-1.57) | 0.007 |
| Obesity | 0.85 (0.74-0.97) | 0.02 | 0.89 (0.73-1.09) | 0.26 |
| Diabetes | 0.68 (0.55-0.85) | 0.001 | 1.25 (0.97-1.62) | 0.08 |
| Pre-frailty/frailty | 0.60 (0.52-0.68) | <0.001 | 1.78 (1.48-2.15) | <0.001 |
| Depression | 0.68 (0.54-0.85) | 0.001 | 1.23 (0.94-1.61) | 0.13 |
| Chronic kidney disease | 0.86 (0.74-0.99) | 0.04 | 0.99 (0.82-1.21) | 0.94 |

RR: risk ratio.

**^a^** Compared to average performers (n=3,492), and additionally adjusted for age, gender and ethnicity.

**^b^** The model compared high performers to average performers (n=5,790).

**^c^** The model compared low performers to average performers (n=4,134).

Note: 1) hypertension was defined as on treatment for high BP or BP >140/90 mmHg at study entry; 2) dyslipidemia was defined as those taking cholesterol-lowering medications or serum cholesterol ≥212 mg/dL (≥5 mmol/L; Australia) and ≥240 mg/dL (≥6.2 mmol/L; U.S.) or LDL > 160 mg/dL (>4.1 mmol/L); 3) obesity was defined as body mass index ≥30; 4) diabetes was defined from self-report or fasting glucose ≥126mg/dL (≥7 mmol/L) or on treatment for diabetes; 5) frailty was defined using the adapted Fried frailty criteria, including being underweight, weak grip strength, exhaustion, slow walking speed and low physical activity, with pre-frail including anyone with 1 or 2 criteria and Frail as anyone with three or more criteria (2); 6) depression was defined as CES-D-10 ≥8; 7) chronic kidney disease was defined as eGFR < 60 ml/min/1.73m2 or urinary albumin to creatinine ratio ≥3 mg/mmol.

**Supplementary Table 6. Associations between education and behaviors, and associated factors, including chronic conditions, when high performers were compared to average performers (N=5,790)**

|  | **Odds ratio (95% CI)** | **P-value** |
| --- | --- | --- |
| Education> 12 years (versus education≤ 12 years) -> |  |  |
| Living alone at home | 0.97 (0.86-1.10) | 0.65 |
| Ever smoker | 1.04 (0.93-1.16) | 0.46 |
| Ever alcohol intake | 1.76 (1.51-2.04) | <0.001 |
| Living alone at home (versus living with someone else) -> |  |  |
| Ever smoker | 1.30 (1.15-1.46) | <0.001 |
| Ever alcohol intake | 1.04 (0.89-1.22) | 0.58 |
| Hypertension | 0.99 (0.87-1.23) | 0.86 |
| Dyslipidemia | 0.86 (0.76-0.98) | 0.02 |
| Diabetes | 1.36 (1.13-1.65) | 0.001 |
| Obesity | 1.10 (0.97-1.25) | 0.13 |
| Pre-frailty/frailty | 1.22 (1.08-1.38) | 0.001 |
| Depression | 1.36 (1.12-1.65) | 0.002 |
| Chronic kidney disease | 1.09 (0.95-1.24) | 0.21 |
| Ever smoker (versus never smoked) -> |  |  |
| Hypertension | 1.08 (0.95-1.22) | 0.23 |
| Dyslipidemia | 1.13 (1.01-1.28) | 0.04 |
| Diabetes | 1.23 (1.02-1.48) | 0.03 |
| Obesity | 1.21 (1.07-1.37) | 0.002 |
| Pre-frailty/frailty | 1.17 (1.04-1.31) | 0.009 |
| Depression | 1.15 (0.95-1.39) | 0.16 |
| Chronic kidney disease | 1.08 (0.95-1.23) | 0.23 |
| Ever alcohol intake (versus never drunk alcohol) -> |  |  |
| Hypertension | 0.86 (0.73-1.02) | 0.08 |
| Dyslipidemia | 1.08 (0.92-1.27) | 0.32 |
| Diabetes | 0.54 (0.43-0.67) | <0.001 |
| Obesity | 0.64 (0.55-0.75) | <0.001 |
| Pre-frailty/frailty | 0.73 (0.63-0.85) | <0.001 |
| Depression | 0.95 (0.74-1.23) | 0.72 |
| Chronic kidney disease | 0.81 (0.69-0.96) | 0.013 |

Note: 1) age, gender and ethnicity were also included in the full model; 2) variables with arrows behind are independent variables, and those below the independent variables are dependent variables; 3) hypertension was defined as on treatment for high BP or BP >140/90 mmHg at study entry; 4) dyslipidemia was defined as those taking cholesterol-lowering medications or serum cholesterol ≥212 mg/dL (≥5 mmol/L; Australia) and ≥240 mg/dL (≥6.2 mmol/L; U.S.) or LDL > 160 mg/dL (>4.1 mmol/L); 5) obesity was defined as body mass index ≥30; 6) diabetes was defined from self-report or fasting glucose ≥126mg/dL (≥7 mmol/L) or on treatment for diabetes; 7) frailty was defined using the adapted Fried frailty criteria, including being underweight, weak grip strength, exhaustion, slow walking speed and low physical activity, with pre-frail including anyone with 1 or 2 criteria and Frail as anyone with three or more criteria (2); 8) depression was defined as CES-D-10 ≥8; 9) chronic kidney disease was defined as eGFR < 60 ml/min/1.73m2 or urinary albumin to creatinine ratio ≥3 mg/mmol.

**Supplementary Table 7. Associations between education and behaviors, and associated factors, including chronic conditions, when low performers were compared to average performers (N=4,134)**

|  | **Odds ratio (95% CI)** | **P-value** |
| --- | --- | --- |
| Education> 12 years (versus education≤ 12 years) -> |  |  |
| Living alone at home | 0.96 (0.84-1.11) | 0.58 |
| Ever smoker | 1.06 (0.93-1.21) | 0.38 |
| Ever alcohol intake | 1.58 (1.33-1.88) | <0.001 |
| Living alone at home (versus living with someone else) -> |  |  |
| Ever smoker | 1.22 (1.06-1.40) | 0.006 |
| Ever alcohol intake | 1.12 (0.94-1.34) | 0.19 |
| Hypertension | 0.93 (0.80-1.10) | 0.41 |
| Dyslipidemia | 0.83 (0.71-0.96) | 0.011 |
| Diabetes | 1.40 (1.14-1.72) | 0.001 |
| Obesity | 1.07 (0.92-1.24) | 0.37 |
| Pre-frailty/frailty | 1.31 (1.14-1.51) | <0.001 |
| Depression | 1.38 (1.11-1.70) | 0.003 |
| Chronic kidney disease | 1.08 (0.93-1.25) | 0.33 |
| Ever smoker (versus never smoked) -> |  |  |
| Hypertension | 1.05 (0.90-1.23) | 0.55 |
| Dyslipidemia | 1.08 (0.94-1.24) | 0.30 |
| Diabetes | 1.19 (0.98-1.46) | 0.08 |
| Obesity | 1.17 (1.01-1.35) | 0.033 |
| Pre-frailty/frailty | 1.24 (1.08-1.41) | 0.002 |
| Depression | 1.11 (0.90-1.38) | 0.32 |
| Chronic kidney disease | 1.14 (0.98-1.32) | 0.09 |
| Ever alcohol intake (versus never drunk alcohol) -> |  |  |
| Hypertension | 0.97 (0.80-1.19) | 0.80 |
| Dyslipidemia | 1.14 (0.95-1.36) | 0.15 |
| Diabetes | 0.58 (0.45-0.73) | <0.001 |
| Obesity | 0.68 (0.57-0.81) | <0.001 |
| Pre-frailty/frailty | 0.73 (0.62-0.87) | <0.001 |
| Depression | 0.88 (0.68-1.14) | 0.33 |
| Chronic kidney disease | 0.78 (0.66-0.94) | 0.009 |

Note: 1) age, gender and ethnicity were also included in the full model; 2) variables with arrows behind are independent variables, and those below the independent variables are dependent variables; 3) hypertension was defined as on treatment for high BP or BP >140/90 mmHg at study entry; 4) Dyslipidemia was defined as those taking cholesterol-lowering medications or serum cholesterol ≥212 mg/dL (≥5 mmol/L; Australia) and ≥240 mg/dL (≥6.2 mmol/L; U.S.) or LDL > 160 mg/dL (>4.1 mmol/L); 5) obesity was defined as body mass index ≥30; 6) diabetes was defined from self-report or fasting glucose ≥126mg/dL (≥7 mmol/L) or on treatment for diabetes; 7) frailty was defined using the adapted Fried frailty criteria, including being underweight, weak grip strength, exhaustion, slow walking speed and low physical activity, with pre-frail including anyone with 1 or 2 criteria and Frail as anyone with three or more criteria (2); 8) depression was defined as CES-D-10 ≥8; 9) chronic kidney disease was defined as eGFR < 60 ml/min/1.73m2 or urinary albumin to creatinine ratio ≥3 mg/mmol.

**Supplementary Table 8. Direct association ^a^ between modifiable factors and cognitive trajectory subgroups, with reference to average performers ^b^ (N=6,432)**

|  | **Highest performers**  **(n=2,298)** | | **Lowest performer**  **(n=642)** | |
| --- | --- | --- | --- | --- |
|  | **Relative RR**  **(95% CI)** | **P-value** | **Relative RR**  **(95% CI)** | **P-value** |
| **Social/lifestyle factors** |  |  |  |  |
| Education> 12 years | 4.64 (4.07-5.29) | <0.001 | 0.71 (0.59-0.85) | <0.001 |
| Living alone at home | 0.89 (0.77-1.02) | 0.09 | 1.26 (1.04-1.52) | 0.02 |
| Ever smoker | 0.90 (0.79-1.03) | 0.12 | 0.92 (0.76-1.11) | 0.41 |
| Ever alcohol intake | 1.39 (1.17-1.66) | <0.001 | 0.79 (0.63-0.99) | 0.04 |
| **Chronic conditions** |  |  |  |  |
| Hypertension | 0.69 (0.60-0.80) | <0.001 | 0.89 (0.72-1.10) | 0.28 |
| Dyslipidemia | 1.06 (0.93-1.22) | 0.36 | 1.30 (1.07-1.57) | 0.007 |
| Obesity | 0.84 (0.73-0.97) | 0.01 | 0.89 (0.73-1.09) | 0.26 |
| Diabetes | 0.69 (0.56-0.86) | 0.001 | 1.27 (0.98-1.64) | 0.07 |
| Pre-frailty/frailty | 0.60 (0.52-0.68) | <0.001 | 1.77 (1.47-2.14) | <0.001 |
| Depression | 0.68 (0.54-0.85) | 0.001 | 1.23 (0.94-1.60) | 0.13 |
| Chronic kidney disease | 0.87 (0.75-1.01) | 0.06 | 1.00 (0.83-1.21) | 0.99 |

RR: risk ratio.

**^a^** Multinomial logistic regression adjusted for age, gender, ethnicity, education, living alone, smoking status, alcohol intake, hypertension, dyslipidemia, obesity, diabetes, frailty status, depression and chronic kidney disease.

**^b^** Compared to average performers (n=3,492).

Note: 1) hypertension was defined as on treatment for high BP or BP >140/90 mmHg at study entry; 2) dyslipidemia was defined as those taking cholesterol-lowering medications or serum cholesterol ≥212 mg/dL (≥5 mmol/L; Australia) and ≥240 mg/dL (≥6.2 mmol/L; U.S.) or LDL > 160 mg/dL (>4.1 mmol/L); 3) obesity was defined as body mass index ≥30; 4) diabetes was defined from self-report or fasting glucose ≥126mg/dL (≥7 mmol/L) or on treatment for diabetes; 5) frailty was defined using the adapted Fried frailty criteria, including being underweight, weak grip strength, exhaustion, slow walking speed and low physical activity, with pre-frail including anyone with 1 or 2 criteria and Frail as anyone with three or more criteria (2); 6) depression was defined as CES-D-10 ≥8; 7) chronic kidney disease was defined as eGFR < 60 ml/min/1.73m2 or urinary albumin to creatinine ratio ≥3 mg/mmol.

**Supplementary Table 9. Direct association ^a^ between modifiable factors and cognitive trajectory subgroups, with reference to average performers ^b^ among Australian and US white participants (N=5,908)**

|  | **Highest performers**  **(n=2,177)** | | **Lowest performer**  **(n=566)** | |
| --- | --- | --- | --- | --- |
|  | **Relative RR**  **(95% CI)** | **P-value** | **Relative RR**  **(95% CI)** | **P-value** |
| **Social/lifestyle factors** |  |  |  |  |
| Education> 12 years | 4.85 (4.24-5.55) | <0.001 | 0.77 (0.64-0.94) | 0.009 |
| Living alone at home | 0.92 (0.80-1.06) | 0.24 | 1.24 (1.01-1.52) | 0.04 |
| Ever smoker | 0.90 (0.78-1.02) | 0.11 | 0.93 (0.76-1.13) | 0.46 |
| Ever alcohol intake | 1.37 (1.14-1.66) | 0.001 | 0.77 (0.60-0.99) | 0.04 |
| **Chronic conditions** |  |  |  |  |
| Hypertension | 0.70 (0.61-0.81) | <0.001 | 0.92 (0.73-1.15) | 0.46 |
| Dyslipidemia | 1.03 (0.89-1.18) | 0.72 | 1.31 (1.07-1.61) | 0.01 |
| Obesity | 0.86 (0.75-0.99) | 0.05 | 0.88 (0.71-1.10) | 0.26 |
| Diabetes | 0.76 (0.60-0.96) | 0.02 | 1.35 (1.02-1.78) | 0.04 |
| Pre-frailty/frailty | 0.63 (0.55-0.73) | <0.001 | 1.73 (1.42-2.11) | <0.001 |
| Depression | 0.68 (0.54-0.87) | 0.002 | 1.27 (0.96-1.68) | 0.10 |
| Chronic kidney disease | 0.90 (0.77-1.05) | 0.18 | 1.02 (0.83-1.25) | 0.87 |

RR: risk ratio.

**^a^** Multinomial logistic regression adjusted for age, gender, education, living alone, smoking status, alcohol intake, hypertension, dyslipidemia, obesity, diabetes, frailty status, depression and chronic kidney disease.

**^b^** Compared to average performers (n=3,165).

Note: 1) hypertension was defined as on treatment for high BP or BP >140/90 mmHg at study entry; 2) dyslipidemia was defined as those taking cholesterol-lowering medications or serum cholesterol ≥212 mg/dL (≥5 mmol/L; Australia) and ≥240 mg/dL (≥6.2 mmol/L; U.S.) or LDL > 160 mg/dL (>4.1 mmol/L); 3) obesity was defined as body mass index ≥30; 4) diabetes was defined from self-report or fasting glucose ≥126mg/dL (≥7 mmol/L) or on treatment for diabetes; 5) frailty was defined using the adapted Fried frailty criteria, including being underweight, weak grip strength, exhaustion, slow walking speed and low physical activity, with pre-frail including anyone with 1 or 2 criteria and Frail as anyone with three or more criteria (2); 6) depression was defined as CES-D-10 ≥8; 7) chronic kidney disease was defined as eGFR < 60 ml/min/1.73m2 or urinary albumin to creatinine ratio ≥3 mg/mmol.

**Supplementary Table 10. Direct association ^a^ between modifiable factors and cognitive trajectory subgroups, with reference to average performers ^b^ by level of education (N=6,432)**

| **Years of education** | **≤ 12 years (n=2,833)** | | | | **> 12 years (n=3,599)** | | | |
| --- | --- | --- | --- | --- | --- | --- | --- | --- |
|  | **Highest performers**  **(n=540, 19.1%)** | | **Lowest performer**  **(n=387, 13.7%)** | | **Highest performers**  **(n=1,758, 49.9%)** | | **Lowest performer**  **(n=255, 7.1%)** | |
|  | **Relative RR**  **(95% CI)** | **P-value** | **Relative RR**  **(95% CI)** | **P-value** | **Relative RR**  **(95% CI)** | **P-value** | **Relative RR**  **(95% CI)** | **P-value** |
| **Social/lifestyle factors** |  |  |  |  |  |  |  |  |
| Living alone at home | 0.96 (0.77-1.21) | 0.75 | 1.22 (0.95-1.57) | 0.11 | 0.86 (0.73-1.02) | 0.08 | 1.32 (0.98-1.77) | 0.07 |
| Ever smoker | 0.87 (0.69-1.09) | 0.21 | 0.91 (0.70-1.17) | 0.45 | 0.93 (0.80-1.09) | 0.36 | 0.94 (0.70-1.25) | 0.65 |
| Ever alcohol intake | 1.72 (1.30-2.27) | <0.001 | 0.75 (0.56-1.01) | 0.06 | 1.23 (0.98-1.54) | 0.08 | 0.82 (0.56-1.19) | 0.29 |
| **Chronic conditions** |  |  |  |  |  |  |  |  |
| Hypertension | 0.76 (0.60-0.97) | 0.03 | 0.94 (0.71-1.24) | 0.65 | 0.64 (0.54-0.76) | <0.001 | 0.83 (0.60-1.15) | 0.25 |
| Dyslipidemia | 1.15 (0.89-1.47) | 0.28 | 1.18 (0.92-1.51) | 0.20 | 1.04 (0.87-1.22) | 0.62 | 1.54 (1.14-2.06) | 0.005 |
| Obesity | 0.83 (0.65-1.05) | 0.11 | 0.91 (0.70-1.17) | 0.46 | 0.85 (0.72-1.01) | 0.06 | 0.83 (0.60-1.15) | 0.27 |
| Diabetes | 0.73 (0.50-1.06) | 0.10 | 1.33 (0.97-1.84) | 0.08 | 0.68 (0.52-0.88) | 0.004 | 1.15 (0.75-1.78) | 0.51 |
| Pre-frailty/frailty | 0.68 (0.54-0.86) | 0.001 | 1.99 (1.56-2.54) | <0.001 | 0.56 (0.47-0.66) | <0.001 | 1.49 (1.11-1.99) | 0.007 |
| Depression | 0.87 (0.60-1.25) | 0.44 | 1.23 (0.86-1.76) | 0.25 | 0.59 (0.48-0.78) | <0.001 | 1.23 (0.82-1.84) | 0.32 |
| Chronic kidney disease | 0.95 (0.74-1.23) | 0.71 | 1.08 (0.84-1.39) | 0.56 | 0.82 (0.69-0.98) | 0.03 | 0.90 (0.67-1.22) | 0.51 |

RR: risk ratio.

**^a^** Multinomial logistic regression adjusted for age, gender, ethnicity, living alone, smoking status, alcohol intake, hypertension, dyslipidemia, obesity, diabetes, frailty status, depression and chronic kidney disease.

**^b^** Compared to average performers (n=3,492).

Note: 1) hypertension was defined as on treatment for high BP or BP >140/90 mmHg at study entry; 2) dyslipidemia was defined as those taking cholesterol-lowering medications or serum cholesterol ≥212 mg/dL (≥5 mmol/L; Australia) and ≥240 mg/dL (≥6.2 mmol/L; U.S.) or LDL > 160 mg/dL (>4.1 mmol/L); 3) obesity was defined as body mass index ≥30; 4) diabetes was defined from self-report or fasting glucose ≥126mg/dL (≥7 mmol/L) or on treatment for diabetes; 5) frailty was defined using the adapted Fried frailty criteria, including being underweight, weak grip strength, exhaustion, slow walking speed and low physical activity, with pre-frail including anyone with 1 or 2 criteria and Frail as anyone with three or more criteria (2); 6) depression was defined as CES-D-10 ≥8; 7) chronic kidney disease was defined as eGFR < 60 ml/min/1.73m2 or urinary albumin to creatinine ratio ≥3 mg/mmol.

**Supplementary Table 11. Direct associations between modifiable factors and cognitive trajectory subgroups, with reference to class 4 (N=16,018)**

|  | **Class 1**  **(2,298, 14.4%)** | | **Class 2**  **(2,777, 17.3%)** | | **Class 3**  **(3,185, 19.9%)** | | **Class 4**  **(3,492 21.8%)** | **Class 5**  **(1,843, 11.5%)** | | **Class 6**  **(1,781, 11.1%)** | | **Class 7**  **(642, 4.0%)** | |
| --- | --- | --- | --- | --- | --- | --- | --- | --- | --- | --- | --- | --- | --- |
|  | RRR (95% CI) | P-value | RRR (95% CI) | P-value | RRR (95% CI) | P-value |  | RRR (95% CI) | P-value | RRR (95% CI) | P-value | RRR (95% CI) | P-value |
| **Social/lifestyle factors** | | | | | | | | | | | | | |
| Education> 12 years | 4.69 (4.14-5.32) | <0.001 | 2.44 (2.20-2.72) | <0.001 | 2.06 (1.86-2.28) | <0.001 | Reference | 1.20 (1.07-1.35) | <0.01 | 0.57 (0.50-0.65) | <0.001 | 0.69 (0.57-0.82) | <0.001 |
| Ever smoker | 0.91 (0.81-1.02) | 0.10 | 0.95 (0.86-1.06) | 0.39 | 0.91 (0.82-1.01) | 0.07 | Reference | 1.08 (0.96-1.22) | 0.21 | 1.03 (0.91-1.17) | 0.65 | 0.92 (0.76-1.10) | 0.37 |
| Ever alcohol intake | 1.45 (1.23-1.71) | <0.001 | 1.32 (1.14-1.53) | <0.001 | 1.11 (0.97-1.27) | 0.14 | Reference | 0.90 (0.77-1.06) | 0.21 | 0.78 (0.67-0.91) | <0.01 | 0.81 (0.64-1.01) | 0.06 |
| Living alone at home | 0.89 (0.78-1.01) | 0.06 | 0.84 (0.75-0.94) | <0.01 | 0.96 (0.86-1.07) | 0.45 | Reference | 1.03 (0.91-1.17) | 0.62 | 1.18 (1.04-1.35) | 0.01 | 1.28 (1.07-1.54) | <0.01 |
| **Chronic conditions** | | | | | | | | | | | | | |
| Hypertension | 0.72 (0.63-0.82) | <0.001 | 0.87 (0.77-0.99) | 0.03 | 0.87 (0.77-0.97) | 0.02 | Reference | 0.91 (0.80-1.05) | 0.20 | 0.96 (0.83-1.11) | 0.56 | 0.86 (0.70-1.06) | 0.17 |
| Diabetes | 0.75 (0.61-0.92) | <0.01 | 0.82 (0.68-0.97) | 0.03 | 0.84 (0.71-0.99) | 0.04 | Reference | 1.15 (0.97-1.37) | 0.11 | 1.20 (1.01-1.43) | 0.04 | 1.24 (0.96-1.59) | 0.09 |
| Obesity | 0.82 (0.72-0.94) | <0.01 | 0.93 (0.83-1.05) | 0.23 | 0.95 (0.85-1.06) | 0.40 | Reference | 0.89 (0.78-1.01) | 0.07 | 1.01 (0.88-1.15) | 0.92 | 0.90 (0.74-1.09) | 0.29 |
| Dyslipidemia | 1.11 (0.98-1.25) | 0.11 | 1.00 (0.90-1.12) | 0.94 | 1.03 (0.93-1.15) | 0.56 | Reference | 1.05 (0.93-1.19) | 0.42 | 1.05 (0.93-1.19) | 0.43 | 1.26 (1.04-1.51) | 0.02 |
| Pre-frailty/frailty | 0.58 (0.51-0.66) | <0.001 | 0.76 (0.68-0.85) | <0.001 | 0.74 (0.67-0.82) | <0.001 | Reference | 0.95 (0.84-1.07) | 0.42 | 1.40 (1.24-1.58) | <0.001 | 1.77 (1.48-2.13) | <0.001 |
| Depression | 0.69 (0.56-0.85) | <0.001 | 0.91 (0.76-1.09) | 0.30 | 0.89 (0.75-1.06) | 0.20 | Reference | 1.09 (0.90-1.32) | 0.36 | 1.19 (0.99-1.43) | 0.07 | 1.26 (0.97-1.63) | 0.08 |
| Chronic kidney disease | 0.88 (0.77-1.01) | 0.06 | 0.88 (0.78-0.99) | 0.03 | 0.91 (0.81-1.02) | 0.11 | Reference | 1.01 (0.89-1.15) | 0.89 | 1.11 (0.97-1.26) | 0.12 | 1.03 (0.85-1.24) | 0.78 |

Note: 1) age, gender and ethnicity were also included in the full model; 2) hypertension was defined as on treatment for high BP or BP >140/90 mmHg at study entry; 3) dyslipidemia was defined as those taking cholesterol-lowering medications or serum cholesterol ≥212 mg/dL (≥5 mmol/L; Australia) and ≥240 mg/dL (≥6.2 mmol/L; U.S.) or LDL > 160 mg/dL (>4.1 mmol/L); 4) obesity was defined as body mass index ≥30; e. diabetes was defined from self-report or fasting glucose ≥126mg/dL (≥7 mmol/L) or on treatment for diabetes; 5) frailty was defined using the adapted Fried frailty criteria, including being underweight, weak grip strength, exhaustion, slow walking speed and low physical activity, with pre-frail including anyone with 1 or 2 criteria and Frail as anyone with three or more criteria (2); 6) depression was defined as CES-D-10 ≥8; 7) chronic kidney disease was defined as eGFR < 60 ml/min/1.73m2 or urinary albumin to creatinine ratio ≥3 mg/mmol.

**Supplementary Table 12. Associations between education and behaviors, and associated factors, including chronic conditions, when the seven trajectory classes were included (N=16,018)**

|  | **Odds ratio (95% CI)** | **P-value** |
| --- | --- | --- |
| Education> 12 years (versus education≤ 12 years) -> |  |  |
| Living alone at home | 0.98 (0.91-1.05) | 0.50 |
| Ever smoker | 1.08 (1.01-1.15) | 0.03 |
| Ever alcohol intake | 1.69 (1.55-1.84) | <0.001 |
| Living alone at home (versus living with someone else) -> |  |  |
| Ever smoker | 1.32 (1.23-1.42) | <0.001 |
| Ever alcohol intake | 1.01 (0.93-1.11) | 0.77 |
| Hypertension | 0.99 (0.92-1.08) | 0.95 |
| Dyslipidemia | 0.92 (0.86-0.99) | 0.04 |
| Diabetes | 1.17 (1.04-1.30) | <0.01 |
| Obesity | 1.10 (1.02-1.19) | 0.01 |
| Pre-frailty/frailty | 1.18 (1.10-1.27) | <0.001 |
| Depression | 1.41 (1.26-1.58) | <0.001 |
| Chronic kidney disease | 1.08 (0.99-1.17) | 0.06 |
| Ever smoker (versus never smoked) -> |  |  |
| Hypertension | 1.11 (1.03-1.19) | <0.01 |
| Dyslipidemia | 1.10 (1.03-1.19) | <0.01 |
| Diabetes | 1.22 (1.09-1.36) | <0.001 |
| Obesity | 1.21 (1.12-1.30) | <0.001 |
| Pre-frailty/frailty | 1.20 (1.12-1.29) | <0.001 |
| Depression | 1.20 (1.07-1.34) | <0.001 |
| Chronic kidney disease | 1.09 (1.01-1.18) | 0.03 |
| Ever alcohol intake (versus never drunk alcohol) -> |  |  |
| Hypertension | 0.85 (0.77-0.94) | <0.001 |
| Dyslipidemia | 1.09 (0.99-1.19) | 0.08 |
| Diabetes | 0.61 (0.54-0.70) | <0.001 |
| Obesity | 0.67 (0.61-0.74) | <0.001 |
| Pre-frailty/frailty | 0.80 (0.73-0.87) | <0.001 |
| Depression | 0.99 (0.86-1.14) | 0.87 |
| Chronic kidney disease | 0.81 (0.73-0.89) | <0.001 |

Note: 1) age, gender and ethnicity were also included in the full model; 2) variables with arrows behind are independent variables, and those below the independent variables are dependent variables; 3) hypertension was defined as on treatment for high BP or BP >140/90 mmHg at study entry; 4) dyslipidemia was defined as those taking cholesterol-lowering medications or serum cholesterol ≥212 mg/dL (≥5 mmol/L; Australia) and ≥240 mg/dL (≥6.2 mmol/L; U.S.) or LDL > 160 mg/dL (>4.1 mmol/L); 5) obesity was defined as body mass index ≥30; 6) diabetes was defined from self-report or fasting glucose ≥126mg/dL (≥7 mmol/L) or on treatment for diabetes; 7) frailty was defined using the adapted Fried frailty criteria, including being underweight, weak grip strength, exhaustion, slow walking speed and low physical activity, with pre-frail including anyone with 1 or 2 criteria and Frail as anyone with three or more criteria (2); 8) depression was defined as CES-D-10 ≥8; 9) chronic kidney disease was defined as eGFR < 60 ml/min/1.73m2 or urinary albumin to creatinine ratio ≥3 mg/mmol.

**Supplementary Table 13. Number of timepoints with complete cognitive data and lengths of follow-up by cognitive trajectory subgroup (N=6,432)**

|  | **Subgroup (N, %)** | | |  |
| --- | --- | --- | --- | --- |
|  | **High performers**  **(2,298, 35.7)** | **Average performers**  **(3,492, 54.3)** | **Low performers**  **(642, 10.0)** | **P-value** |
|  | **Median (inter-quartile range)** | | | |
| **Number of cognitive assessments** |  |  |  |  |
| 3MS | 4 (3-4) | 3 (3-4) | 3 (2-4) | <0.001 |
| COWAT | 4 (3-4) | 3 (3-4) | 3 (2-4) | <0.001 |
| HVLT-R delayed recall | 4 (3-4) | 3 (3-4) | 3 (2-3) | <0.001 |
| SDMT | 4 (3-4) | 3 (3-4) | 3 (2-4) | <0.001 |
| **Length of follow-up (years)** |  |  |  |  |
| 3MS | 4 (3-5) | 4 (3-5) | 3 (1-5) | <0.001 |
| COWAT | 4 (3-5) | 3 (3-5) | 3 (1-5) | <0.001 |
| HVLT-R delayed recall | 4 (3-5) | 3 (3-5) | 3 (1-4) | <0.001 |
| SDMT | 4 (3-5) | 3 (3-5) | 3 (1-4) | <0.001 |

3MS, Modified Mini-Mental State Examination; COWAT-F, Controlled Oral Word Association Test-F; HVLT-R, Hopkins Verbal Learning Test–Revised (delayed recall); SDMT, Symbol Digit Modalities Test; P-values are based on Kruskal–Wallis rank test.

**Supplementary Figure 1.** **Joint trajectory plots of individual cognitive tests (N=17,724).** The x-axis denotes the year of cognitive assessment at baseline as well as 1, 3, 4, 5, 6 and 7 years of follow-up. The y-axis denotes the raw scores of the cognitive tests. The percentages refer to the proportions of participants assigned into the corresponding classes. 3MS, Modified Mini-Mental State Examination; COWAT-F, Controlled Oral Word Association Test-F; HVLT-R, Hopkins Verbal Learning Test–Revised (delayed recall); SDMT, Symbol Digit Modalities Test.

**
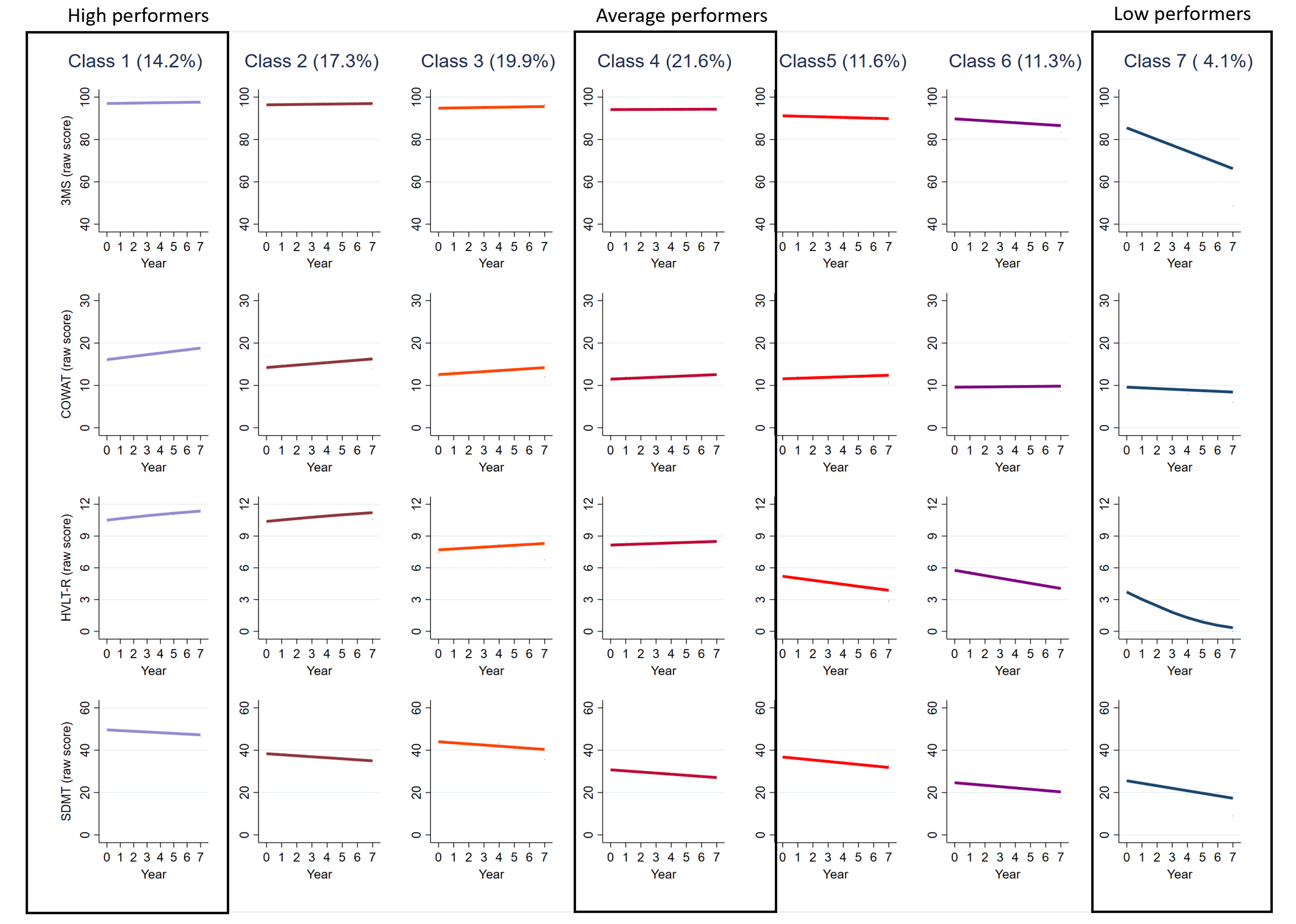
**

**Supplementary Figure 2. Strategies to promote high cognitive performance and prevention of low cognitive performance in older community-dwelling individuals** *** Smoking was not directly associated with cognitive trajectories, but was indirectly associated with both high and low cognitive performance via chronic conditions. ** Living alone was only directly associated with low cognitive performance, but was indirectly associated with both high and low cognitive performance via chronic conditions.**


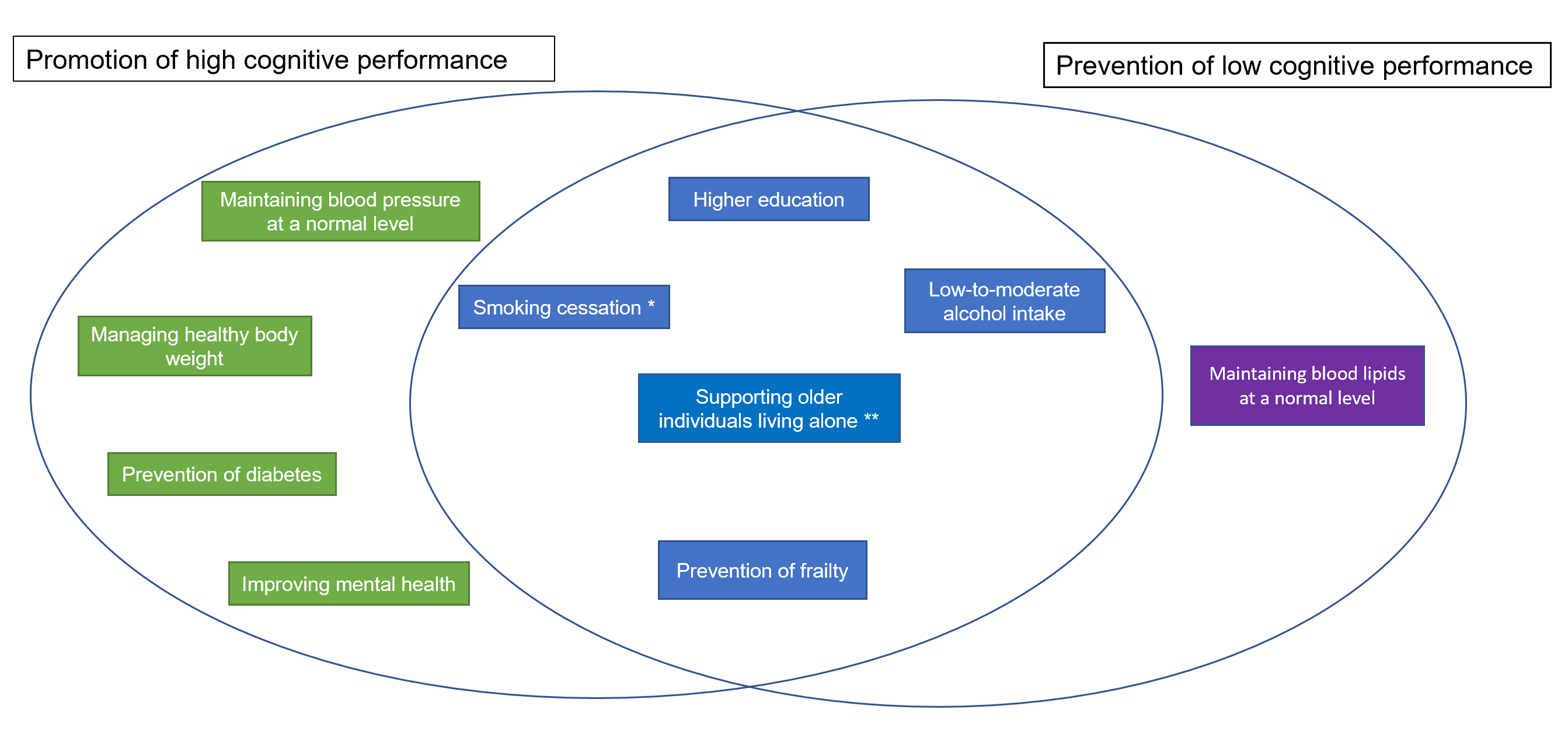


**REFERENCE**

1. Aspree Investigator Group. Study design of ASPirin in Reducing Events in the Elderly (ASPREE): a randomized, controlled trial. Contemp Clin Trials. 2013;36(2):555-64.

2. Fried LP, Tangen CM, Walston J, Newman AB, Hirsch C, Gottdiener J, et al. Frailty in older adults: evidence for a phenotype. J Gerontol A Biol Sci Med Sci. 2001;56(3):M146-56.
